# Comparative studies of catalytic pathways for *Streptococcus pneumoniae* sialidases NanA, NanB and NanC

**DOI:** 10.1038/s41598-018-38131-z

**Published:** 2019-02-15

**Authors:** Kela Xiao, Xingyong Wang, Haibo Yu

**Affiliations:** 1 School of Chemistry and Molecular Bioscience, University of Wollongong, Wollongong, NSW 2500 Australia; 2 Molecular Horizons, University of Wollongong, Wollongong, NSW 2500 Australia; 3 Illawarra Health and Medical Research Institute, Wollongong, NSW 2500 Australia

## Abstract

*Streptococcus pneumoniae* (*S*. *pneumoniae*) is a leading human pathogen, which takes large responsibility for severe otitis media, acute meningitis and septicaemia. It encodes up to three distinct sialidases: NanA, NanB and NanC, which are promising drug targets. Recent experimental studies have shown that these three sialidases might work together up to the ultimate step, where NanA and NanB produce *N*-acetylneuraminic acid (Neu5Ac) and 2,7-anhydro-Neu5Ac following the functions of sialidase and intramolecular *trans*-sialidase, whilst NanC carries on a ping-pong mechanism that produces or removes 2-deoxy-2,3-didehydro-Neu5AC. It is intriguing that these sialidases have similar active sites but operate via three distinct reaction pathways. To clarify this issue, herein we present the first systematic computational investigation on the catalytic pathways for *S*. *pneumoniae* NanA, NanB and NanC based on combined quantum mechanics/molecular mechanics simulations, and propose the most preferred routes for the three *S*. *pneumoniae* sialidases. Our findings support the mechanisms of NanA and NanC that were proposed by previous experimental studies, whereas the role of water in NanB was found to differ slightly from our current understandings. The mechanistic insights obtained from this work are expected to assist in the design of potent inhibitors targeting these key enzymes for therapeutic applications.

## Introduction

*S*. *pneumoniae* (pneumococcus) is a Gram-positive, *α*-haemolytic, facultative anaerobic organism that resides in the nasopharynx. *S*. *pneumoniae* is largely responsible for upper/lower-respiratory infections, meningitis, and septicaemia, and operates in a number of fairly major diseases with a considerably high mortality and incidence^1^. The occurrence rate of life-threatening pneumococcal disease in human, especially in children under 5 years of age is higher than HIV, malaria, and measles combined^2^. It has been shown that *S*. *pneumoniae* encodes up to three distinct neuraminidases (NAs, also called sialidases): NanA, NanB and NanC^3–5^. Clinical research of pneumococcal isolates of *S*. *pneumoniae* points out that these three sialidases are present in 100%, 96% and 51% of strains, respectively^3^. NanA and NanB have been shown to play a crucial role in respiratory tract infection and sepsis^1^. Moreover, considerable evidences indicate that NanA is essential to middle ear invasion, nasopharynx and the incidence of otitis media effusion^6^. *S*. *pneumoniae* sialidases have been shown to have great potential as drug targets or vaccine candidates^7^. Recent studies have shown that the human upper respiratory epitheliums express *α* 2,6-sialylated glycans while many human bronchial epithelial cells possess *α* 2,3-linked sialic acid substrates^8,9^. NanA would mainly be responsible for the sialic acid removal^4^, while NanB would be essential for the survival of the colonised bacteria^4,9^, and NanC was suggested to be a regulator of NanA^4,10^. All these findings indicate that the three *S*. *pneumoniae* sialidases might play distinct roles in pneumococcal virulence.

**Figure 1:**
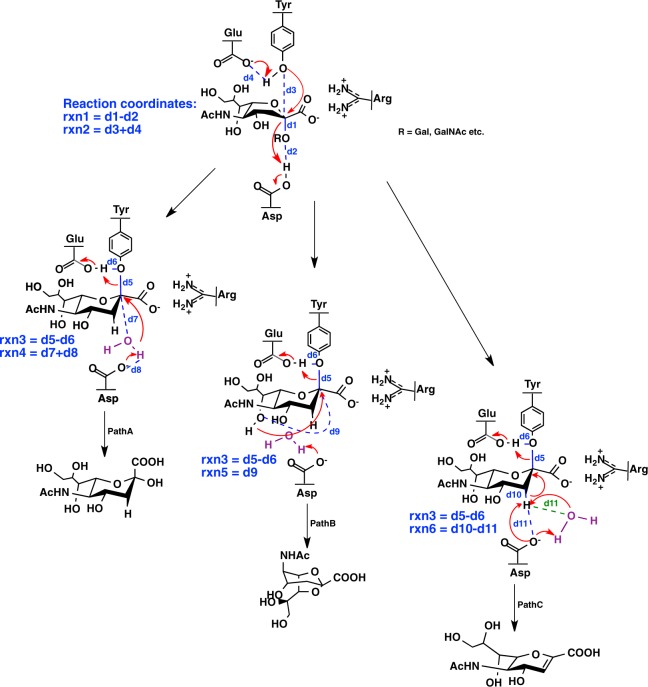
The three predefined pathways for NanA, NanB and NanC. For the first step, d1 refers to the distance between C2 position of the sugar ring and the glycosidic O of methyl group, d2 is the distance between the glycosidic O of methyl group and the aspartic acid H*δ*2, d3 describes the distance between the tyrosine O*η* and C2 position of the sugar ring, and d4 is the one between the tyrosine H*η* and the glutamic acid O*ε*2. For the second steps, d5 is the distance between the tyrosine O*η* and C2 position of the substrate, d6 corresponds to the one between the tyrosine O*η* and H*η*, d7 is the one between C2 position of the sugar ring and the catalytic water oxygen, d8 is the one between aspartic acid residue O*ε*2 and catalytic water hydrogen, d9 defines the one between C2 position and O7 of the sugar ring, and d10 refers to the one between C3 and H32 in substrate. For NanB and NanC, d11 (labelled in blue) accounts for that between H32 of the sugar ring and the aspartic residue Oε2. However, for NanA, due to the reaction is proceeded with an activated water molecule nearby because the more accessible the catalytic cavity in NanA allows more water molecules to reside, d11 (labelled in green) is defined by the distance between H32 of the sugar ring and catalytic water oxygen. The reaction coordinates rxn1-rxn6 are also illustrated in the figure. Tyr, Glu, Asp: Tyr752, Glu647, Asp372 in NanA; Tyr653, Glu541, Asp270 in NanB; Tyr695, Glu584, Asp315 in NanC.

Sequence identity between NanB and NanC is the greatest (over 50%), while it is limited between NanA and NanB (up to 24%). The crystal structures of NanA, NanB and NanC have been solved and demonstrate conservation of their catalytic domains^10–13^. Previous studies^12^ have suggested NanA to be a typical hydrolytic sialidase that cleaves *α* 2,3-, *α* 2,6- and *α* 2,8-linked sialic acids, and produces *N*-acetylneuraminic acid (*α*-Neu5Ac). NanB acts as an intramolecular (IT) *trans*-sialidase that preferentially cleaves *α* 2,3-linked sialic acid substrates to release 2,7-anhydro-Neu5Ac^12,13^. NanC is shown to be specific for α 2,3-linked sialic acids, producing the primary product 2-deoxy-2,3-didehydro-N-acetyl- neuraminic acid (Neu5Ac2en) that can be hydrolysed to Neu5Ac by attacking C2 of the oxocarbonium ion^10^. The reaction mechanisms for the three sialidases NanA, NanB and NanC in *S*. *pneumoniae* have been proposed by previous experimental studies based on X-ray crystallography, nuclear magnetic resonance (NMR) spectroscopy, kinetic analysis and site-directed mutagenesis (Fig. 1)^4,10–15^. For the conserved catalytic residues shared by these three sialidases, the carboxylate group of sialic acids interacts with the tri-arginine clusters, and the tyrosine residue close to the glutamic acid acts as a nucleophile, while an aspartic residue acts as an acid/base (Fig. 1). The first step shows a common reaction pathway, whereas the second step exhibits intriguing differences. The C2 position of the oxocarbonium ion is attacked by a water molecule, the sialyl cation O7 atom, and the carbohydrate acceptor for NanA, NanB and NanC, respectively. Subsequent products (Neu5Ac, 2,7-anhydro-Neu5Ac and Neu5Ac2en) are then formed. The general picture is consistent with other retaining glycosidases^16^. It is further supported by the captured sialyl-enzyme covalent intermediate on the conserved tyrosine residue in trans-sialidase TcTS and the bacterial sialidase *C*. *perfringens* NanI^17–19^. The experimental findings^4,10^ indicated that these three sialidases might work together up to the ultimate step, where NanA and NanB produce Neu5Ac and 2,7-anhydro-Neu5Ac following the functions of sialidase and IT *trans*-sialidase, whilst NanC could carry on a ping-pong mechanism that produces or removes Neu5Ac2en.

It is intriguing that these three different sialidases have similar active sites but operate via three distinct reaction pathways^4^. To fully understand the molecular factors dictating their respective catalyses, we explored the hydrolysis mechanisms of NanA, NanB and NanC by systematically comparing the energy profiles of all possible reaction pathways on the basis of combined quantum mechanics/molecular mechanics (QM/MM) simulations. Based on their structural and sequence homology to other well-studied (trans)salidases, it is believed that their first steps share a common reaction pathway, while the second steps present intriguing differences which produce three different products, supported by NMR and thiobarbituric assay studies^4^. Therefore, we focus on the reaction barriers for the second step. The most preferred routes have been determined for all three NAs. In addition, based on the combined QM/MM simulations of the intermediate state of NanA, a detailed analysis on the active site structures and dynamics including water has been carried out. These computational results, together with previous experimental findings, thus provide a better understanding of the details on the catalytic mechanisms of these three *S*. *pneumoniae* sialidases.

## Results and Discussions

### Equilibrated structures from MM and QM/MM simulations of RC

**Figure 2:**
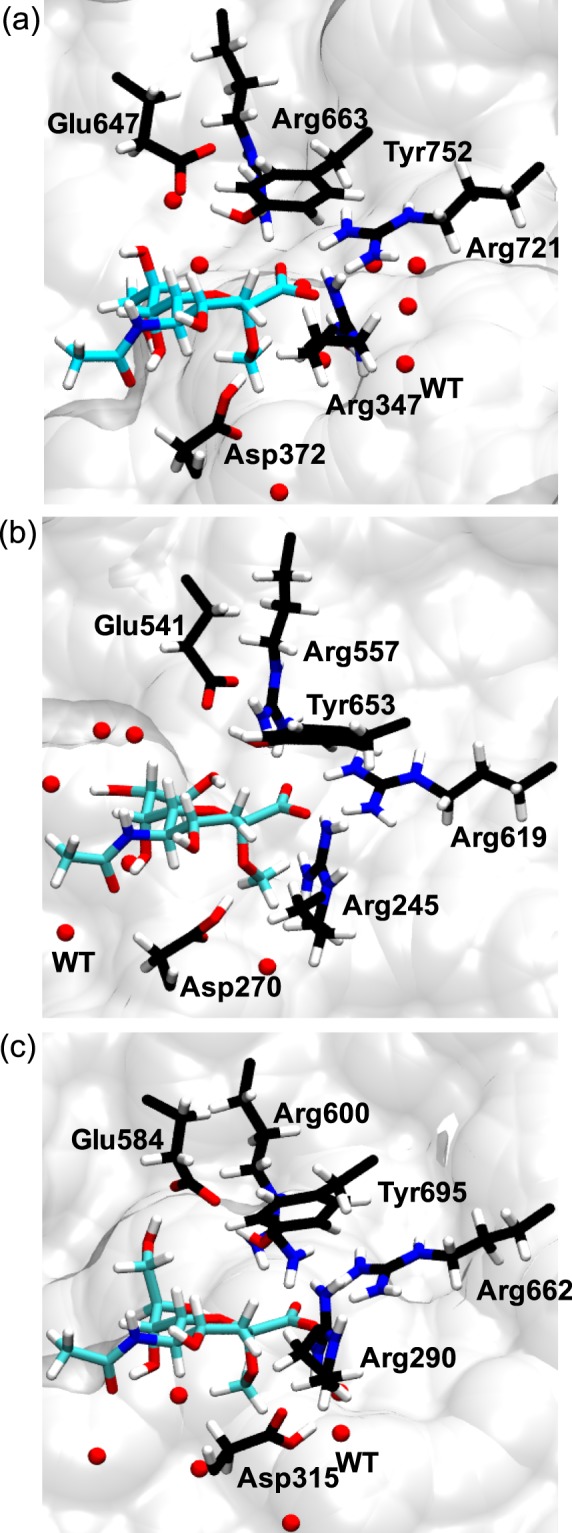
The final snapshots from the explicit solvent SCC-DFTB/MM simulations. The key residues and substrate are shown explicitly. The aliphatic hydrogen atoms are omitted for clarity. The water molecules within approximately 5 Å of O*ε*2 of Glu647, Glu541 and Glu695 are shown in red, respectively. (**a**) NanA; (**b**) NanB; (**c**) NanC.

The equilibrated structures of the reactant complexes (RC) from SCC-DFTB/MM simulations are shown in Fig. 2. The aspartic acid residues (NanA, Asp372; NanB, Asp270; NanC, Asp315) are protonated, and the glutamic acids (NanA, Glu647; NanB, Glu541; NanC, Glu584) are deprotonated in RC. The sugar rings are in a boat conformation $({}^{2}B_{5})$. For the key residues and substrates, the heavy atom positional root-mean-square-deviations (RMSD) from the crystal structures were approximately 1.2 Å over the simulation time. Also, classical MM simulations were carried out for RC to probe the key interactions. Encouragingly, both MM and SCC-DFTB/MM simulations provide a consistent structure that is suitable for the study of the hydrolysis mechanism. The final snapshots from these QM/MM simulations (Supporting Information Table 1) were taken as the initial structures for minimal energy pathway (MEP) calculations.

### Potential energy profiles for NanA, NanB and NanC along three different reaction pathways

We assumed that NanA, NanB and NanC could produce three promiscuous products by following three distinct reaction pathways. A comparative minimal energy pathway (MEP) simulation was performed along the hypothetical reaction pathways presented in Fig. 1. The reaction coordinates used to drive the reactions are also shown in Fig. 1.

#### The first step in NanA, NanB and NanC

**Figure 3:**
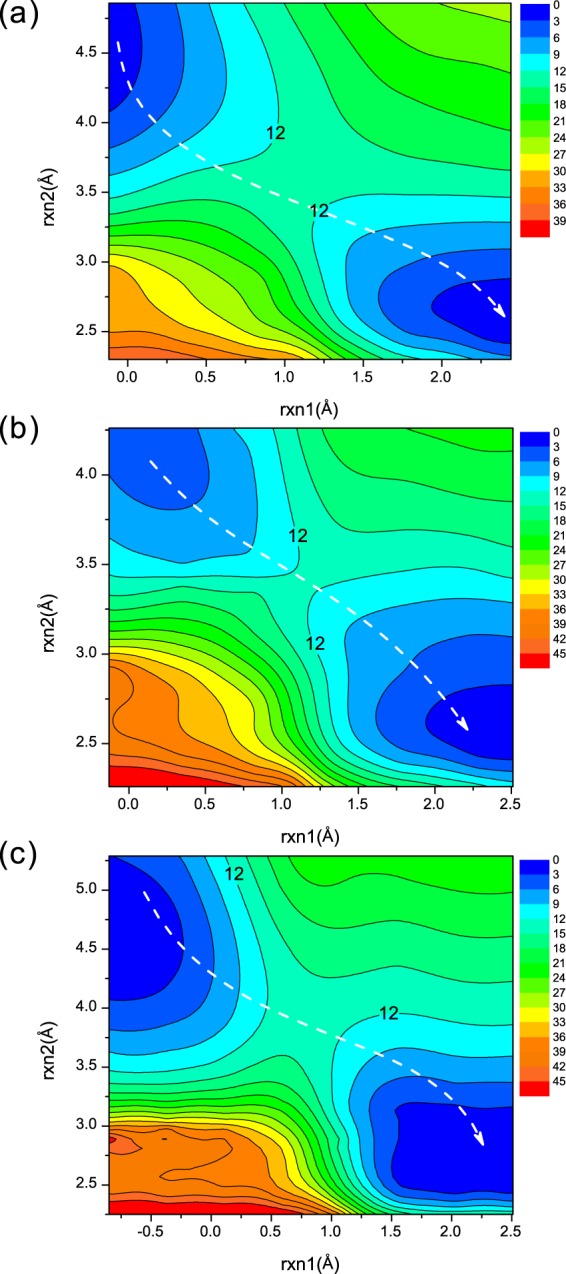
Potential energy contour plot for the first reaction step of (**a**) NanA, (**b**) NanB, and (**c**) NanC. Energies are in kcal/mol. The white dashed line illustrates the minimum potential energy path.

The MEP calculations were carried out along those predefined reaction coordinates. Iterative cycles scanning from the reactant state to the product state forward and backward were performed to achieve convergence. For the first step of NanA, NanB and NanC, rxn1 was harmonically restrained from −0.12 to 2.44 Å, −0.13 to 2.51 Å and −0.85 to 2.5 Å respectively, and rxn2 was scanned from 4.86 to 2.30 Å, 4.34 to 2.26 Å and 5.29 to 2.33 Å, respectively. These two reaction coordinates drive the nucleophilic attack to the anomeric carbons of the substrates and the proton transfer from tyrosines to glutamic acids (NanA, Tyr752 and Glu647; NanB, Tyr653 and Glu541; NanC, Try695 and Glu584). The scans were constructed with a harmonic force constant of 2,000.0 kcal/mol/Å^2^ in steps of 0.08 Å. A total number of 257, 209 and 347 energy points have been obtained in the 2D MEP calculations for the first step in NanA, NanB and NanC, respectively. The contour plots of the energy profiles are shown in Fig. 3, describing the reaction pathways from RC to the intermediate complex (IC) in NanA, NanB and NanC. The white dashed lines highlighted the minimum energy paths that estimate the most likely pathways. Regarding the energetics, the reaction barriers for the first steps are 13.4, 12.3 and 12.5 kcal/mol for NanA, NanB and NanC, respectively, and the path is downhill to the IC once the transition complex of the first reaction step (TS1) is reached, indicating a nucleophilic attack to the anomeric carbon of the substrate and a proton transfer from tyrosines to glutamic acids (NanA, Tyr752 and Glu647; NanB, Tyr653 and Glu541; NanC, Try695 and Glu584).

#### The second step in NanA, NanB and NanC

Subsequently equilibrium SCC-DFTB/MM simulations were performed on the IC structure obtained from the MEPs of the first step (Supporting Information Table 1). Similarly, iterative cycles MEP scanning were performed based on the equilibrated IC structures until convergence was achieved. We investigated the second steps of all the three pathways (PathA, PathB and PathC) for NanA, NanB and NanC, respectively. The setup for reaction coordinates rxn3 to rxn6, as well as the total number of energy points scanned, are listed in Supporting Information Table 2. The scans were conducted with a 0.08 Å step using a harmonic force constant of 2,000.0 kcal/mol/Å^2^.

**Figure 4:**
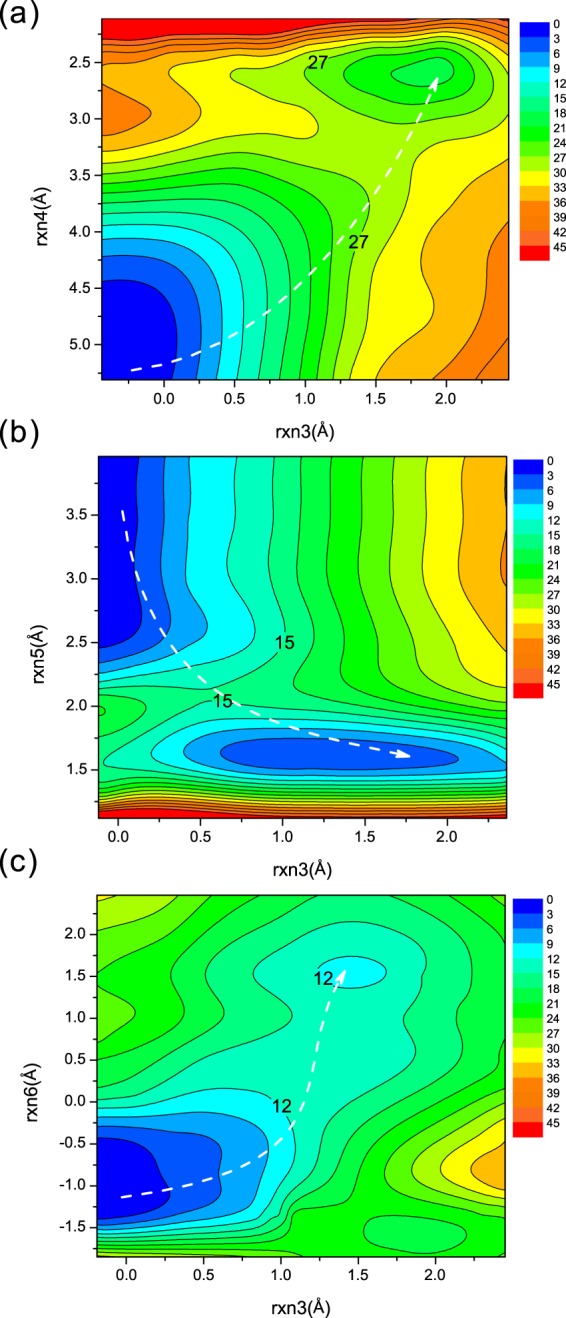
Potential energy contour plot for the second step along (**a**) PathA in NanA, (**b**) PathB in NanB, and (**c**) PathC in NanC. Energies are in kcal/mol. The white dashed line illustrates the minimum potential energy path.

**Figure 5:**
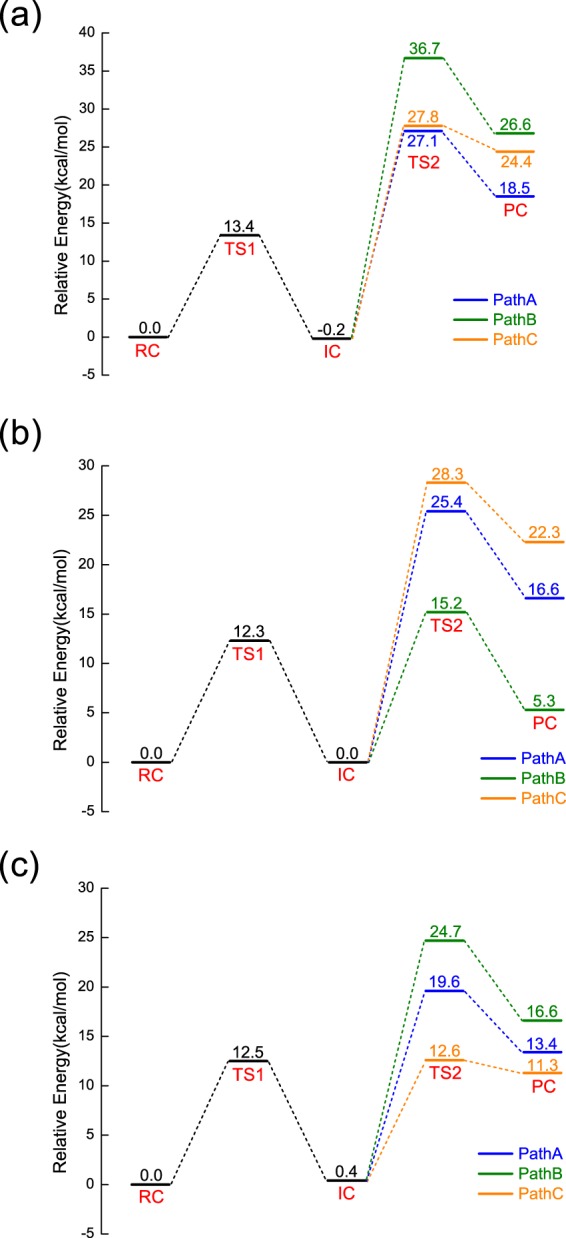
Potential energy profiles (in kcal/mol) for (**a**) NanA, (**b**) NanB, and (**c**) NanC along different reaction pathways.

The potential energy profiles for the second steps along PathA (PathB/PathC) of NanA (NanB/NanC) are shown in Fig. 4 and Supporting Information Fig. 3. The white dashed lines display the minimum energy paths from IC to the transition complexes involved in the second steps (TS2) and then to the product complex (PC). The related reaction barriers for each path of NanA, NanB and NanC are summarised in Fig. 5. It is evident that PathA for NanA, PathB for NanB and PathC for NanC are most favourable, which is consistent with previous proposals. Interestingly, the reaction barriers difference between PathA and PathC for NanA is not large (~0.7 kcal/mol), implying PathC might be a possible pathway for NanA while PathA is more thermodynamically favourable (18.5 kcal/mol vs 24.4 kcal/mol, Fig. 5a).

It is worth noting that PathB for NanB slightly differs from that proposed in the literature^4,10^, in which a proton relay through a catalytic water is involved. However, our simulations indicate an alternative pathway (Fig. 5b) without a water is more favourable than that with a catalytic water (for comparison see Supporting Information Fig. 9). This can be rationalised by the relatively hydrophobic environment of its active site (Fig. 2).

### Description of the mechanisms for NanA, NanB and NanC from the reactant state to the product state

**Figure 6:**
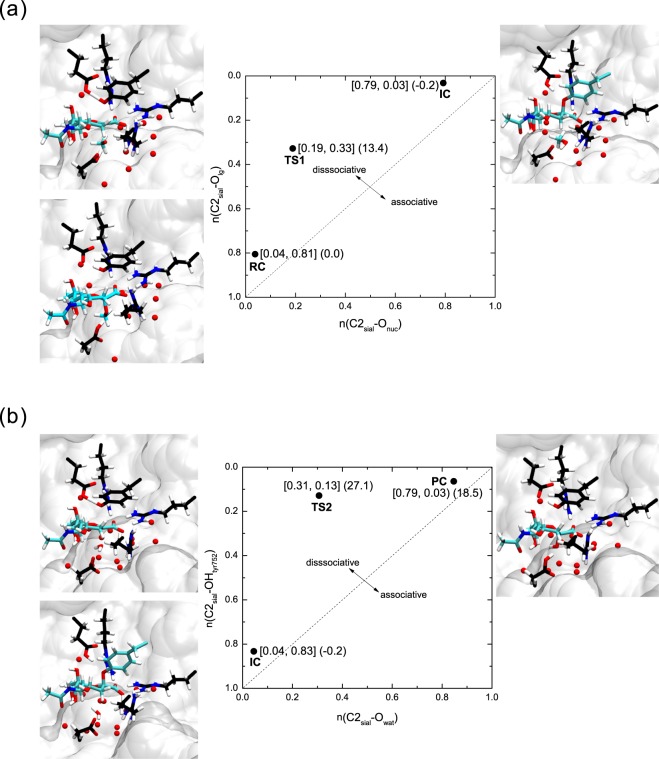
The More O’Ferrall-Jencks plot for the (**a**) IC and (**b**) PC formation of NanA. The axes are the Pauling bond orders from the anomeric C to the nucleophile(n(C2sial-Onuc)) and the leaving group (n(C2sial-Olg)). The RC, TS1, and IC configurations are shown in the bottom left, top left, and top right, respectively. See Fig. 2 for more information. The water molecules are shown in red with CPK.

The MEPs for the three different reaction pathways, PathA, PathB and PathC of NanA, NanB and NanC all proceed diagonally, indicating an A_N_D_N_ type mechanism. This represents a concerted bimolecular “SN2” displacement^20^, where the reaction coordinates rxn1 and rxn2, rxn3 and rxn4, rxn3 and rxn5, rxn3 and rxn6 are highly correlated. The A_N_D_N_ reaction can be classified into either an associative or a dissociative mechanism according to the relationship between attacking and departing groups, such as the nucleophilic attack by tyrosine (Tyr752 in NanA, Tyr653 in NanB and Tyr695 in NanC) and the departure of the methyl group in the first step. The 2D More O’Ferrall-Jencks style diagram^21^ was depicted, aiming to provide relevant information regarding the mechanistic scenario for the IC and TS formations which has been proved to be a controversial subject of the catalytic mechanism of glycosidases^22^. In addition, from the 2D More O’Ferrall-Jencks style diagram, we can gain useful information about their hydrolysis mechanisms related to their different reaction pathways (e.g. associative or dissociative A_N_D_N_ mechanism, and how they achieve their different PCs through different TS structures). The Pauling bond order^23^ was used to represent the sialic acid bond making and breaking, which was determined as follows

1 $${n}_{x}={n}_{0}\exp [\frac{({R}_{0}-{R}_{x})}{0.6}]$$

where *R*_0_ is the reference bond length (*R*_0_ equals to 1.4 Å and 1.09 Å for C–O and C–H, respectively), whose bond order is defined as *n*_0_ (=1), and *R*_x_ denotes the instantaneous distance between two atoms of interest. Fig. 6a and Supporting Information Figs 18–19 present the More O’Ferrall-Jencks plots for the critical points for the formation of IC during the sialidase activity of NanA, NanB and NanC. The axes represent the Pauling bond orders for the tyrosine (Tyr752 in NanA, Tyr653 in NanB and Tyr695 in NanC) O*η*–sialyl bond making and O (leaving group)–sialyl bond breaking. It is clear that the leaving methyl group departs substantially when the TS1 is reached, indicating that the first steps of the three systems follow a dissociative A_N_D_N_ mechanism.

The axes in the More O’Ferrall-Jencks plots for PC formation (Fig. 6b and Supporting Information Figs 18–19) denote the Pauling bond orders from C2 at the axial position to the catalytic water or the tyrosine residues (Tyr752 in NanA, Tyr653 in NanB and Tyr695 in NanC). For all the three cases along PathA (6b and Supporting Information Figs 18–19), the tyrosine O*η*–sialyl bond has already been cleaved while the O (water)–sialyl bond formation is beginning, indicating that the hydrolysis reaction of the IC along PathA involves a dissociate A_N_D_N_ mechanism. Similarly, for the three systems in PathB, the tyrosine O*η*–sialyl bond has been cleaved before the formation of the O7 (sialyl)–C2 (sialyl) bond (Supporting Information Fig. 19). However, unlike the reaction along PathB of NanA, where a proton has transferred from the catalytic water to the Asp372 while the water molecule is protonated by HO7 of the sialyl cation (Supporting Information Figure 18), no catalytic water molecules were observed to be involved in the reactions of NanB and NanC. Interestingly, the HO7 of sialyl cation is transferring or has transferred to the aspartic acid residues (Asp270 in NanB and Asp315 in NanC). Supporting Information Figs 18–19 reveal that in PathC, the tyrosine O*η*-sialyl bond is broken before TS2 is reached. At this point, the proton transfer between the catalytic water molecule and the Asp372 of NanA is about to occur, and the proton transfer between the aspartic acid residue (Asp270 in NanB and Asp315 in NanC) and H32 of the sialyl cation has happened. Taking into account all of the above results, it can be concluded that the A_N_D_N_ mechanisms involved in all pathways are dissociative, indicating that the loss of bonding in the TS has progressed further than bond making. This dissociative mechanism has been observed in the computational study of the catalytic mechanisms of TcTS and TrSA^24,25^. It is worth noting that the proton transfers between the glutamic acids and the tyrosines are not fully completed at the PC states along all the pathways. The subsequent reactions are provided in Supporting Information Figs 18–19. We will focus on the energy profiles shown above in the following context as we are particularly interested in the different nucleophilic attacks at the C2 position of the sialic acid along the three reaction pathways and the underlying molecular factors.

The values for the distances at the critical points are presented in (Supporting Information Tables 3–8). At TS1, for all the three systems, the proton transfer between the tyrosine and the glutamic acid residue is almost completed (d4 ≈ 1.18 Å, 1.14 Å and 1.21 Å, respectively), and the tyrosine hydroxyl oxygen is moving closer to the anomeric carbon of sialic acid. The tyrosines in the three cases are more negatively charged, which enhances their nucleophilicity. Unlike the traditional carboxylate nucleophile, it has been argued that Tyr752 here offers an easier electrostatic approach to the anomeric carbon of sialic acid due to the more favourable electrostatic interaction with the negatively charged substrate^19,24,26^. The distance from the hydroxyl oxygen of the nucleophilic tyrosine to the C2 position of the substrate in NanB is shorter than those in NanA and NanC by 0.16 and 0.40 Å, respectively, making Tyr653 of NanB a better nucleophile (see details in Supporting Information Table 6). Additionally, the tyrosine O*η*–sialyl has already been cleaved (d1 ≈ 2.07 Å, 2.24 Å and 2.10 Å, respectively) and the aspartic acid residue proton has transferred to the methyl group (d2 ≈ 1.22 Å, 1.05 Å and1.02 Å, respectively). Significant conformational changes of the sugar ring have been observed during the first reaction step. The sialic acid adopts a ${}^{2}B_{5}$ conformation in RC and changes to ${}^{4}H_{5}$ half-chair conformation at the oxocarbenium ion-like TS1, and finally reaches a ${}^{2}C_{5}$ chair form at IC. This itinerary is consistent with the proposed mechanisms based on theoretical studies of other retaining (trans)sialidase^17,27,28^.

In the IC state for NanA, a stable hydrogen bond is formed between the catalytic hydrogen and Asp372 O*ε*2. The average distance between the anomeric carbon and the oxygen atom of the catalytic water molecule is less than 4 Å (d7 ≈ 3.26 Å) and the average angle for hydroxyl oxygen (Tyr752)–C2(sialic acid)–O(water) is ≈178°, providing an ideal position for a nucleophilic attack by a water molecule and the subsequent proton transfer. In contrast, in the IC state for NanB and NanC, the distances between C2 position of the sugar ring and the catalytic water oxygen are 4.59 Å and 5.08 Å, respectively. This is due to the more hydrophobic pockets surrounding the catalytic clefts of NanB and NanC. At TS2 in PathA, the tyrosine–sialic acid bonds have been cleaved (d5 ≈ 2.63 Å, 2.83 Å and 2.47 Å, respectively), while the proton transfers between the glutamic acids and the tyrosines are about to happen (d6 ≈ 1.15 Å, 1.21 Å and 1.22 Å, respectively). This proton transfer is also observed at TS2 in PathC when the O7 (sialyl)-C2 (sialyl) bond formation is beginning in PathB.

### Free energy profiles for NanA along three different paths

**Figure 7:**
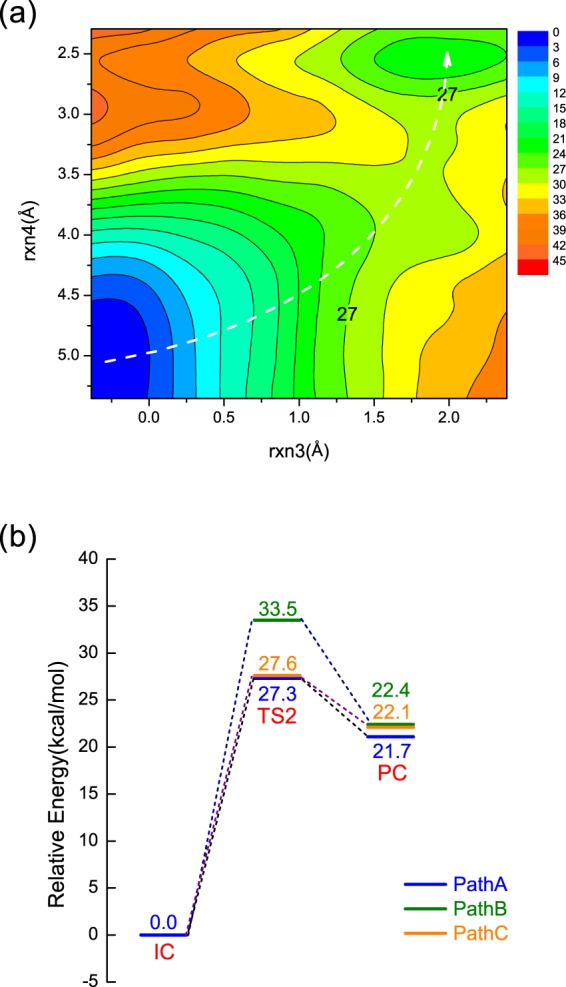
(**a**) Free energy contour plot for PathA of NanA. Energies are in kcal/mol. The white dashed line illustrates the minimum free energy path; (**b**) Free energy profiles for PathA (blue), PathB (green) and PathC (orange) of NanA.

To better understand the chemical reaction for the second step of NanA, the potential of mean force (PMF) simulations have been carried out using the same reaction coordinates based on the structures obtained from the above MEP simulations. Figure 7a and Supporting Information Fig. 20 show the free energy contour plots for the three different reaction pathways from IC to PC and Fig. 7b summarises the reaction energetics from IC to PCs based on the PMF calculations. The minimum free energy paths highlighted with white dashed lines show similar patterns with those obtained from the MEP calculations, which validates the catalytic mechanisms of NanA obtained from MEP calculations. Besides, the MEP and PMF reaction barriers are close to each other, with the largest difference being less than 3.5 kcal/mol. Based on the MEP results, it was concluded that the second step is the rate-limiting step (Fig. 5). Our PMF simulations predicted an activation barrier of 27.3 kcal/mol along PathA for NanA (Fig. 7).

We note that the barrier of 27.3 kcal/mol is higher for a typical enzymatic system^29^. However, this result is similar to those reported for other retain GHs including *S*. *marcescens* ChiB (25.8 kcal/mol)^30^, TcTS (26.8 kcal/mol)^25^, 1,3-1,4-*β*-glucanase (32.0 kcal/mol)^31^ and Golgi *α*-mannosidase II (GMII, 23.0 kcal/mol)^32^. Previous experimental studies showed that the catalytic efficiency of NanA is higher than NanB and NanC^4,12,33^. This is inconsistent with our results that NanA has a higher reaction energy barrier (Fig. 5). It has been suggested in the literature that the carbohydrate-binding module (CBM) can greatly affect the catalytic efficiency of large sialidases^34^. However, we only focus on the catalytic domains of NanA, NanB and NanC with the GSBP setup in our simulations, bringing considerable approximations during the calculations. No structural studies have been performed on the interactions between the CBM40 domains and the substrates of NanA, thus it is envisaged that CBM40 domains of NanA may have the biggest effect on its catalytic efficiency. Additionally, previous work has shown that different buffers, pH optimum and substrates can also affect the enzymatic activity of sialidases^33,34^. On the other hand, it has been noted that SCC-DFTB/MM overestimates the reaction barriers for the classical reaction pathways in glycoside hydrolases^35,36^, likely due to the intrinsic limitation of the SCC-DFTB method. Future work with high level DFT or *ab initio* method is required to reproduce quantitatively the observed kinetic data. Nevertheless, our current study qualitatively reproduced the observed mechanistic difference for three sialidases.

### What are the major determinants for their distinct catalytic pathways?

We carried out perturbative analyses on the effects of residues of interest on the activation barrier (see more details in Supporting Information Section 11). We focused on the different residues among NanA, NanB and NanC, which are thought to be the main determinants for their distinct catalytic pathways. In the perturbative analysis, their partial charges were set to zero and the activation barrier was recalculated based on the corresponding optimised structures of the wild type^26,37^. In all the cases, the resultant activation barriers are relatively similar to that of PathA in the wild type (Supporting Information Table 9), which indicated that these residues have relatively minor effects on the reaction energetics. However, it is worth noting that these residues are relatively far away from the reactive centre and their effects might be largely screened by the environment.

**Figure 8:**
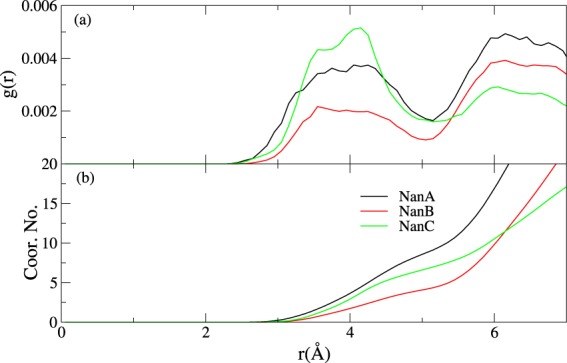
(**a**) Radial distribution function of water oxygens around C2 in the substrate in the IC states of NanA, NanB and NanC; (**b**) The coordination number of water around C2 in the substrate in the IC states of NanA, NanB and NanC.

Instead, we further hypothesised that the local water distribution around the active site plays a more significant role. We characterised this by looking at the water distribution in their respective IC simulations (Fig. 8). For NanA, the overall values of the coordination number for the water molecules around C2 position of the substrate are much higher than those for NanB and NanC. This means that in the IC, the C2 of the sugar in NanA is more exposed to water, and this is consistent with the hypothesis that the water molecule can easily attack C2 position in NanA. Previous analyses of their structural properties in the intermediate states further support this hypothesis (see Description of the Mechanisms for NanA, NanB and NanC). In summary, the differences in their sequence/structure at the active site will likely introduce substantial differences in their local solvation environment, which then contributes to their preferred catalytic pathways. This observation will have significant implications for designing TS analogues as potent inhibitors. For instance, TS analogues with different substitutions around the C7 position of the sialyl cation of NanB (hydrophobic vs hydrophilic) will likely affect their binding affinities. In return, this will help to (dis)approve the hypothesis regarding the local environment in the active sites.

## Conclusions

In this work, the detailed catalytic mechanisms for three closely related *S*. *pneumoniae* sialidases were studied. Based on the comparative MEP simulations, the complete pathways from the reactant state to the product state were characterised. It was confirmed that NanA and NanC operate via the mechanisms consistent with those proposed in the literature. In contrast, the mechanism for NanB is slightly different from the previously proposed one, which involves a catalytic water in the second step. Our simulations strongly suggested that the local hydration environment in the active site strongly favours an alternative mechanism without a catalytic water molecule in the second step. Combining all the data together, we suggest the lowest reaction barriers for the most preferred PathA in NanA, PathB in NanB and PathC in NanC, respectively. It was noted that very different solvation environments in the intermediate states might play a crucial role in the determination of their preferred reaction pathways. Additionally, analyses of the transition states and intermediate states indicate that all three enzymes operate through a dissociative A_N_D_N_ mechanism. The detailed characterisation of the TS complex will provide insights into inhibitor design.

## Methods

### Simulation setup

The crystal structures of NanA (PDB code: 2VVZ^11^, resolution 2.50 Å), NanB (PDB code: 2VW1^12^, resolution 2.39 Å) and NanC (PDB code: 4YW3^10^, resolution 2.05 Å) were used as the starting structures for all the simulations. The protonation states of all the titratable residues, particularly the catalytic residues of NanA (Glu647, Ty752 and Asp372), NanB (Glu541, Tyr653 and Asp270) and NanC (Glu584, Tyr695 and Asp315), were predicted based on the empirical method PROPKA^38^ which has been proved to be capable of providing satisfactory p*K*_a_ predictions and assigning proper protonation states in our previous work^37^. The Glu647, Glu541 and Glu584 were predicted to be deprotonated, while Asp372, Asp270 and Asp315 were shown to be protonated in the Michaelis complex (reactant complex RC). This is consistent with their catalytic roles either as a general base (Glu) or a general acid (Asp) in the first step from RC to the Intermediate complex (IC). All QM/MM simulations were carried out with CHARMM (version c38a2)^39^. CHARMM force field c36^40^ was used to describe the protein and sugars. The self-consistent charge density functional tight binding (SCC-DFTB)^28,41,42^ was adopted to describe the quantum region. The choice of SCC-DFTB as the QM method in our QM/MM simulations is based on the following considerations. Firstly, SCC-DFTB/MM simulations have been proved to be one of the most popular tools to study enzymatic reactions thanks to their efficiencies^42^. Secondly, SCC-DFTB/MM or DFTB3/MM simulations have been successfully applied to study other sialidases^24,25^. The link atom was placed between C*β* and C*γ*, C*β* and C*γ*, and C*β* and C*α* for the glutamic acid, tyrosine and aspartic residue (Glu647, Tyr752 and Asp372 in NanA; Glu541, Tyr653 and Asp270 in NanB; Glu584, Tyr695 and Asp315 in NanC), respectively. Notably, the R group in the substrate was modified to a methyl group considering the computational cost.

The system was solvated with the TIP3P water model^43^. The General Solvent Boundary Potential (GSBP)^44^ setup was used in conjunction with explicit water molecules in the inner sphere for the QM/MM simulation that includes: (i) a spherical region of 20 Å radius centred on the glutamic acid (Glu647 in NanA, Glu541 in NanB and Glu584 in NanC) O*ε*2 was treated as the inner region and the remaining protein is treated as the outer region; (ii) a spherical region of radius 18 Å was defined as the reaction region where Newtonian dynamics was solved; (iii) the region between 18 Å and 20 Å was treated as buffer region where Langevin dynamics is solved. A non-polar cavity potential was adopted to confine the inner region water molecules and prevent them leaving this region. The dielectric constants in the protein and solvent were assumed to be 1.0 and 80.0, respectively. The simulated temperature was set up to 298.15 K. The bonds involving hydrogen were constrained via the SHAKE algorithm^45^. To treat the bonded interactions at the QM/MM boundary, the bonds between the QM and MM regions were cut by a link atom with the divided frontier charge model (DIV)^46^. Such a treatment has been shown to provide a balanced description of proton affinities for molecules of interest^26^. Further more, the puckering of the sugar was converted to a boat conformation $({}^{2}B_{5})$ based on the Cremer-Pople puckering coordinates^47^ during the QM/MM minimisation for consistency with the conformation in the proposed catalytic mechanism^28^. This was achieved by applying a harmonic restraint on the reaction coordinates with the predefined reference values. The whole substrate was also included in the QM region. Full QM/MM minimisation was performed prior to MD simulations. Supporting Information Table 1 summaries the relevant information for the starting structures and equilibrium molecular dynamics simulations. We refer to Supporting Information Section 1 and 2 for more information.

### The minimum energy pathway (MEP) calculations

A comparative MEP simulation was performed along the hypothetical reaction pathways presented in Fig. 1. The reaction coordinates used to drive the reactions are also shown in Fig. 1. In the MEP calculations, the adapted basis, Newton-Raphson approach, is used for energy minimisation, with a gradient threshold of 0.05 kcal/mol/Å for the last several (≈4) adiabatic mapping cycles^48^. The transition states were then identified by locating the potential energy maxima along these approximate reaction paths.

#### From RC to IC

From RC to IC, the three reaction pathways share the same reaction coordinates. rxn1 (d1 − d2) describes the departure of the leaving group. rxn2 (d3 + d4) represents the proton transfer from tyrosine to glutamic acid and the nucleophilic attack on the C2 position of the sugar ring by the deprotonated tyrosine. d1 is the distance between the C2 position of the sugar ring and the glycosidic O of the methyl group. d2 describes the distance between the glycosidic O of the methyl group and the aspartic acid residue (NanA, Asp372; NanB, Asp270; NanC, Asp315) H*δ*2. d3 refers to the distance between the tyrosine (NanA, Tyr752; NanB, Tyr653; NanC, Tyr695) O*η* and C2 position of the sugar ring, and d4 represents the distance between the tyrosine H*η* and the glutamic acid O*ε*2 (NanA, Tyr752 and Glu641; NanB, Tyr653 and Glu541; NanC, Tyr695 and Glu584).

#### From IC to PC

During the second step (from IC to the Product complex PC), the reaction is driven by 2D reaction coordinates. rxn3 defines the cleavage of the tyrosine–sialic acid bond and the protonation of tyrosine by a proton of the glutamic acid. d5 refers to the distance between the tyrosine O*η* and C2 position of the sugar ring, and d6 corresponds to the one between the tyrosine O*η* and H*η* (NanA, Tyr752; NanB, Tyr653; NanC, Tyr695). rxn4, rxn5 and rxn6 define the nucleophilic attack on C2 position of the sugar ring with the deprotonated catalytic water molecule by the aspartic acid residue (PathA), or O7 of the sialyl cation and the carbohydrate acceptor (PathB and PathC), respectively (Glu647, Tyr752 and Asp372 in NanA; Glu541, Tyr653 and Asp270 in NanB; Glu584, Tyr695 and Asp315 in NanC).

PathA: rxn3 = d5 − d6 and rxn4 = d7 + d8 were used to define the reaction pathway from IC to PC Neu5Ac (PathA). d7 is the distance between the C2 position of the sugar ring and the catalytic water oxygen, and d8 is the one between the aspartic acid residue (NanA, Asp372; NanB, Asp270; NanC, Asp315) O*ε*2 and the catalytic water hydrogen.

PathB: rxn3 = d5 − d6 and rxn5 = d9 were used to describe the pathway from IC to PC 2,7-anhydro-Neu5Ac (PathB). d9 defines the distance between C2 position and O7 of the sugar ring.

PathC: rxn3 = d5 − d6 and rxn6 = d10 − d11 were used to describe the one from IC to PC Neu5Ac2en (PathC). d10 refers to the distance between C3 and H32 in substrate. d11 is the distance between H32 of the sugar ring and O*ε*2 of the aspartic acid residue for NanB and NanC (NanB, Asp270; NanC, Asp315) but between H32 of the sugar ring and catalytic water oxygen for NanA.

When simulating PathC in NanA, unlike the catalytic mechanism for PathC in NanC (Supporting Information Fig. 4) proposed by previous studies, in which the C3 proton of the substrate is attacked directly by the catalytic base Asp315^4,10^, the reaction in our setup proceeds with an activated water molecule nearby because the more accessible catalytic cavity in NanA allows more water molecules to reside in (Fig. 1). This mechanism is more accordant with the actual environment in NanA. In contrast, due to the similarity between NanB and NanC in the catalytic mechanisms, in which the distance between the anomeric carbon C2 and aspartic acid residue is rather short and the pocket around the substrate is hydrophobic^4,10^, the reaction involved in the second steps of PathC for NanB were undertaken without activated water molecules nearby (Fig. 1). The water distributions around C2, HO7 and H32 of the substrate were rationalised during the simulations. In comparison, we performed MEP calculations on the reaction path for the second step of NanA based on the mechanism of NanC (See details in Supporting Information Section 5). We also carried out MEP calculations on the reaction paths for the second steps of NanB and NanC based on the mechanisms that involve a catalytic water molecule (See details in Supporting Information Section 7).

### The potential of mean force (PMF) calculations

In this work, we performed PMF simulations on the second step of NanA and compared the difference between the MEP and PMF reaction barriers to probe the effect of thermal fluctuations and flexibilities. The PMF simulations were carried out with a harmonic force constant 50.0 kcal/mol/Å^2^ for each reaction coordinate except 350 kcal/mol/Å^2^ for rxn5. The total number of windows was around 266, 980 and 931 for PathA, PathB and PathC, respectively. For each window, 200 ps SCC-DFTB/MM simulations following the same GSBP protocol were performed. The 2D Weighted Histogram Analysis Method (WHAM) method was used to unbias the systems and to evaluate the free energy profiles along the predefined reaction coordinates^49,50^.

## Supplementary information

Supplementary Info Xiao et al.
