# Supplementary material for: Comparative studies of catalytic pathways for Streptococcus pneumoniae sialidases NanA, NanB and NanC
Source: Sci Rep. 2019 Feb 15;9:2157. doi: 10.1038/s41598-018-38131-z (PMC6377674; doi:10.1038/s41598-018-38131-z)
Supplement: Supplementary file 1 — Supplementary Info Xiao et al. [file 41598_2018_38131_MOESM1_ESM.pdf]

Supporting Information: Comparative studies of catalytic pathways for Streptococcus pneumoniae sialidases NanA, NanB and NanC

Kela Xiao,[†,‡] Xingyong Wang,[†,‡] and Haibo Yu[*,†,‡,¶]

†*School of Chemistry and Molecular Bioscience, University of Wollongong, NSW 2500, Australia*

‡*Molecular Horizons, University of Wollongong, NSW 2500, Australia*

¶*Illawarra Health and Medical Research Institute, Wollongong, Australia*

E-mail: hyu@uow.edu.au

Phone: +61 (2) 4221 4235. Fax: +61 (2) 4221 4287

1 QM/MM simulation setup with the GSBP model

To illustrate the QM/MM simulation setup, we described the procedure for setting up NanA simulations in more details here. The GSBP[1] setup was carried out in conjunction with explicit water molecules in the inner sphere for the QM/MM simulation that includes (Fig. 1): (i) a spherical region of 20 Å radius centred on Glu647 Oϵ2 was described as the inner region and the remaining part was outer region represented with the continuum electrostatics; (ii) a spherical model of 18 Å centred on Glu647 Oϵ2, where Newton's equations of motion were solved (Reaction region); (iii) the region between reaction and inner region, where Langevin dynamics was solved (Buffer region). The link atom was placed between C_β and C_γ, C_β and C_γ, and C_β and C_α for Glu647, Tyr752 and Asp372 respectively to divide the bond between

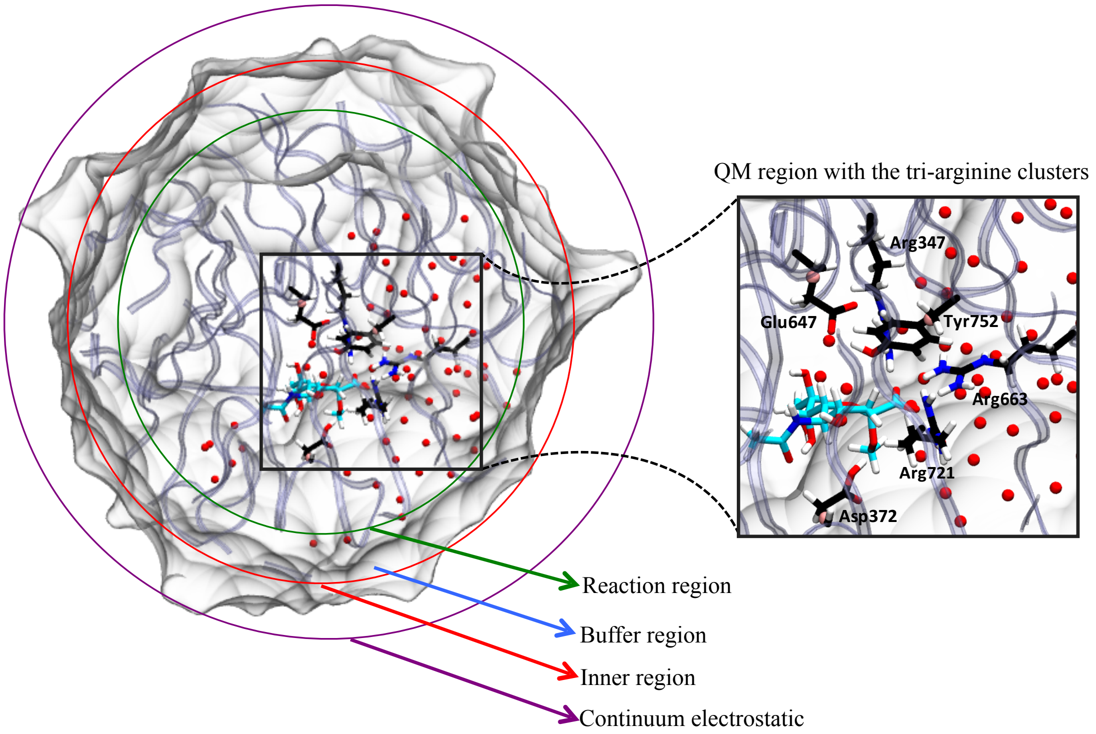

Figure 1: QM/MM simulation setup with the GSBP model for NanA. The inner region, reaction region and buffer region is shown in red, green and blue, respectively; the link atom is drawn in light orange.

QM and MM region (Fig. 1). The QM atoms are include C_γ, Hγ1, Hγ2, C_δ, Oδ1, Oδ2 of Glu647, C_γ, C_δ1, Hδ1, C_δ2, Hδ2, C_ϵ1, Hϵ1, C_ϵ2, Hϵ2, C_ζ, OHη, Hη of Tyr752, C_β, Hβ1, Hβ2, C_γ, Oγ1, Oγ2, Hγ2 of Asp372 and the whole substrate.

2 The sugar puckering of the substrate in the reactant complex (RC)

The puckering of the sugar was converted to a boat conformation ($^2B_5$) (Fig. 2) based on the Cremer-Pople puckering coordinates[2,3] during the QM/MM minimization for consistency with the conformation in the proposed catalytic mechanism. The puckering coordinates

were given by Q, ϕ and θ defined by Cremer and Pople.[2] This was achieved by applying harmonic restraints with a force constant 1.0, 50.0 and 50.0 kcal/mol, respectively, around the predefined reference values.

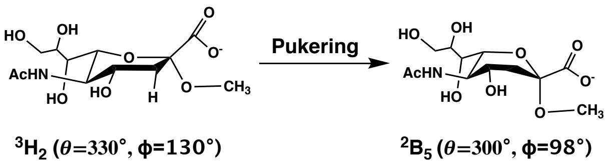

Figure 2: The proposed conformation of the substrate in the RC.

3 Summary of the equilibrium molecular dynamics simulations for RC and IC of NanA, NanB and NanC

All the simulated systems (RC: reactant complex and IC: intermediate complex) are summarised in Table 1.

Table 1: Summary of equlibrium molecular dynamics simulartions of RC and IC of NanA, NanB and NanC.

System		PDB ID	No of QM atoms[b]	MM (ns)	QM/MM (ns)
NanA	RC	2VVZ[4a]	70	100	4
	IC	2VVZ[4]	67		4
NanB	RC	2VW1[5a]	70	100	4
	IC	2VW1[5]	67		4
NanC	RC	4YW3[6a]	70	100	4
	IC	4YW3[6]	67		4

[a] In the simulations, the inhibitors (DAN: 2-deoxy-2,3-dehydro-N-acetyleneuraminic acid) were converted the natural substrates; [b] The SCC-DFTB/MM partition boundary (the link atom) for all the simulations was placed between C_β and C_γ, C_β and C_γ, and C_β and C_α in residue Glu647, Tyr752 and Asp372 of NanA, in residue Glu541, Tyr653 and Asp270 of NanB, and in residue Glu584, Tyr695 and Asp315 of NanC, respectively. The DIV scheme was adopted as it has been shown to provide the best representation of the system.[7]

4 Potential energy profiles for the second steps in NanA, NanB and NanC

Table 2: Summary of the setup for reaction coordinates rxn3 to rxn6 (in Å) and the total number of structures scanned for the second step along the three paths of NanA, NanB and NanC.

System		rxn3	rxn4 in PathA<br>rxn5 in PathB<br>rxn6 in PathC	No. of structures
NanA	PathA	-0.44–2.44	5.29–2.11	317
	PathB	-0.28–2.12	3.91–1.03	211
	PathC	-0.30–2.10	-1.73–2.11	319
NanB	PathA	-0.14–2.50	6.66–2.10	421
	PathB	-0.12–2.36	3.96–1.12	251
	PathC	-0.25–2.55	-1.95–2.53	442
NanC	PathA	-0.03–2.45	6.87–2.07	402
	PathB	-0.19–2.45	4.12–1.10	302
	PathC	-0.19–2.45	-1.85–2.47	385

Iterative MEP scanning were performed based on the equilibrated IC structures until the convergences occurred. For the second steps along PathA in NanA, NanB and NanC, rxn3 was scanned from -0.44 to 2.44 Å, -0.14 to 2.50 Å and from -0.03 to 2.45 Å while rxn4 was restrained from 5.29 to 2.11 Å, 6.66 to 2.10 Å and from 6.87 to 2.07 Å, respectively.For the second steps along PathB for the three cases, rxn3 was scanned from -0.28 to 2.12 Å, -0.12 to 2.36 Å and from -0.19 to 2.45 Å while rxn5 was sampled from 3.91 to 1.03 Å, 3.96 to 1.12 Å and from 4.12 to 1.10 Å separately. for the second step along PathC, rxn3 was scaned from -0.30 to 2.10 Å, -0.25 to 2.55 Å and from -0.19 to 2.45 Å while rxn6 was scanned from -1.73 to 2.11 Å, -1.95 to 2.53 Å and from -1.85 to 2.47 Å, respectively. The scans were done with a 0.08 Å step with a harmonic force constant of 2,000 kcal/mol/Å^2. The total number of energy points for the second step along PathA, PathB and PathC of NanA is 317, 211 and 319, respectively, and 421, 251 and442 energy points for NanB and 402, 302 and 385 energy points for NanC were obtained for the second steps along the three distinct pathways (Table 2).

The potential energy profiles for the second steps along PathB/PathC of NanA, PathA/PathC of NanB and PathA/PathB of NanC are shown in Fig. 3. The white dashed lines in the figures show the minimum energy paths from IC to the transition complexes involved in the second steps (TS2) and then to PC. These energetic properties can be compared with their preferred pathways shown in Fig. 4 in the maintext.

5 MEP calculations for the second step of NanA along the alternative reaction path of PathC without a catalytic water

The 2D energy profile on the reaction path of the second step for NanA based on the mechanism of NanC[6] was evaluated with two predefined reaction coordinates, rxn3=d5-d6 and rxn7=d10-d11 (Fig. 4). This reaction pathway differs from the PathC investigated in Fig. 1 in the maintext and Fig. 3, which has a catalytic water molecule involved. d5 refers to the distance between Tyr752 Oη and C2 position of the sugar ring, d6 corresponds to the one between Tyr752 Oη and Hη, d10 refers to the one between C3 and H32 in substrate, and d12 accounts for that between H32 of the sugar ring and Asp372 Oϵ2 (Fig. 4). rxn3 was harmonically restrained from -0.22 to 2.02 Å while rxn7 was scanned from -1.63 to 1.53 Å using a force constant of 2,000.0 kcal/mol/Å^2. The scans were constructed in steps of 0.08 Å. A total of 280 energy points have been obtained inthe 2D MEP calculations. The contour plot of the energy profiles is shown in Fig. 5. The energy barrier is around 43.8 kcal/mol which is significantly higher than the one for PathC in NanA studied in Fig. 3 (≈28.0 kcal/mol). This is a further proof that NanA can notproduce Neu5Ac along the reaction path of NanC, no matter whether there is a catalytic water.

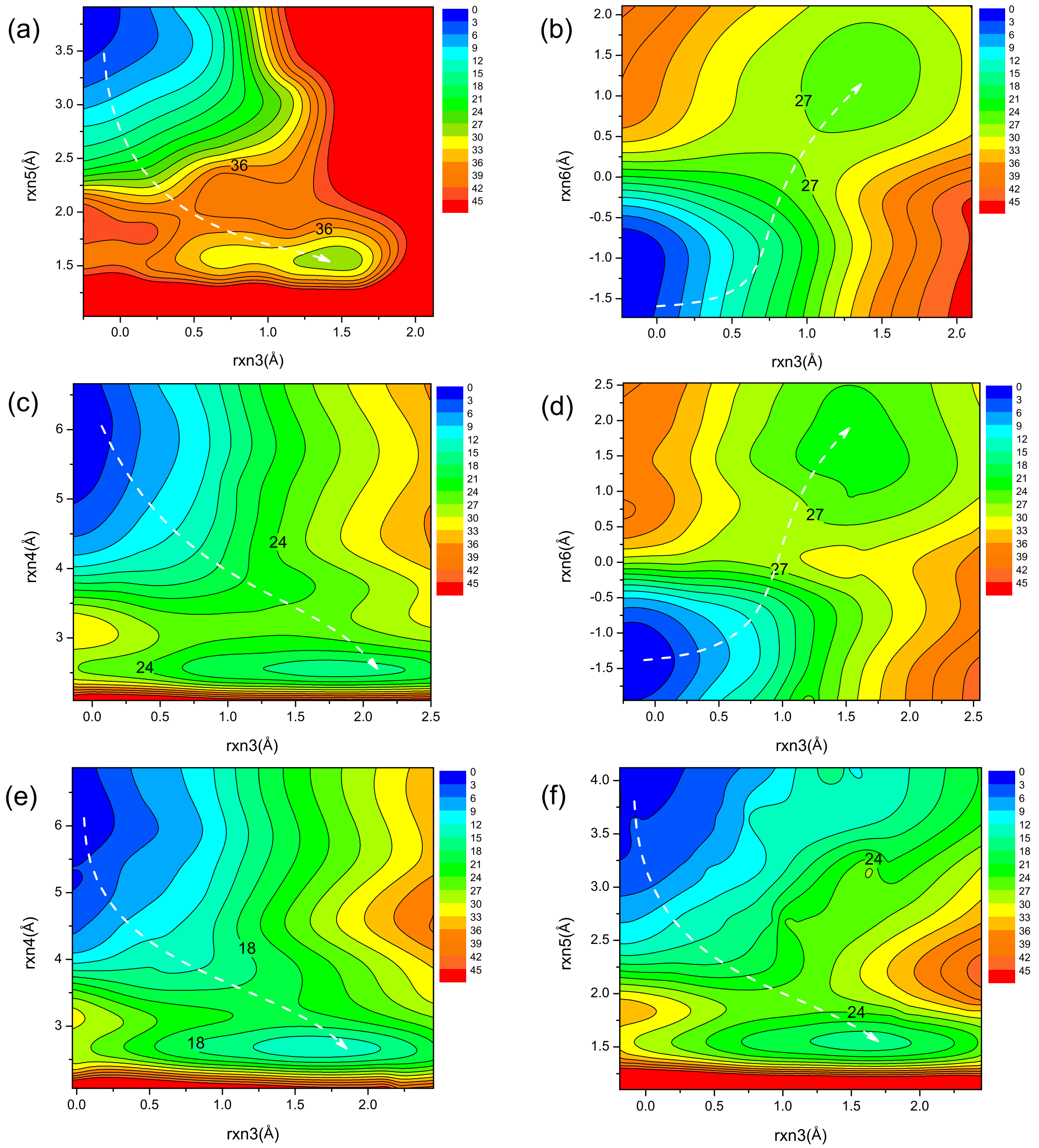

Figure 3: Potential energy contour plots for the second step along (a) PathB in NanA, (b) PathC in NanA, (c) PathA in NanB, (d) PathC in NanB, (e) PathA in NanC,and (f) PathB in NanC. Energies are in kcal/mol. The white dashed line illustrates the minimum potential energy path.

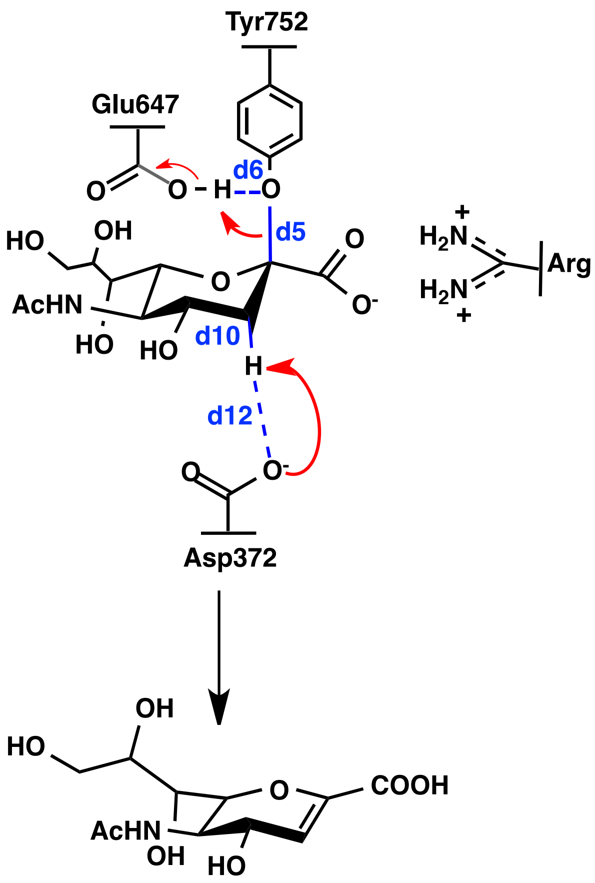

Figure 4: The alternative mechanism for the second step of NanA along PathC. Note that there is no catalytic water involved.

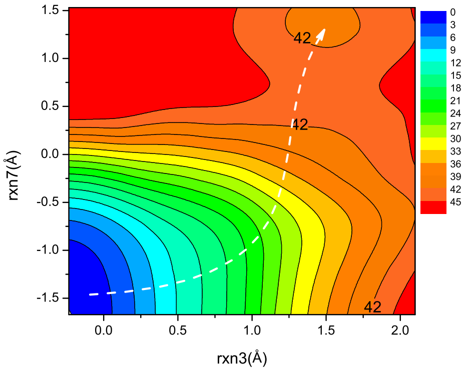

Figure 5: Potential energy contour plot for the second step of NanA based on the mechanism proposed for NanC. Energies are in kcal/mol.

6 The extension MEP calculations for PathB and PathC of NanA to reach the final PC state

As the proton transfers between the Tyr752 and Glu647 involved in both PathB and PathC of NanA have not been fully completed in their PC states, we expanded the MEP calculations

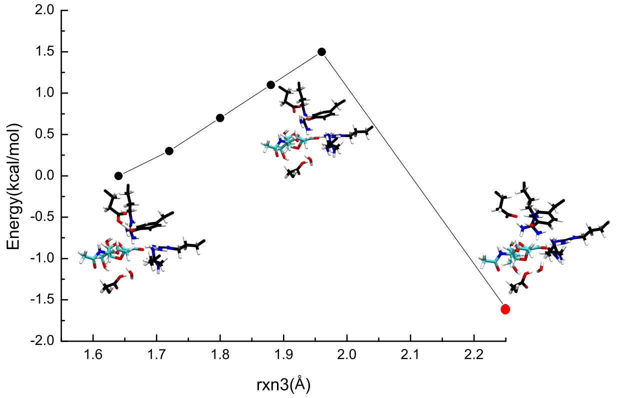

Figure 6: 1D potential energy plot (in kcal/mol) of the subsequent reaction for PathB in NanA. The last point (labelled in red) represents a free minimisation without restraints on the reaction coordinates to locate the PC state.

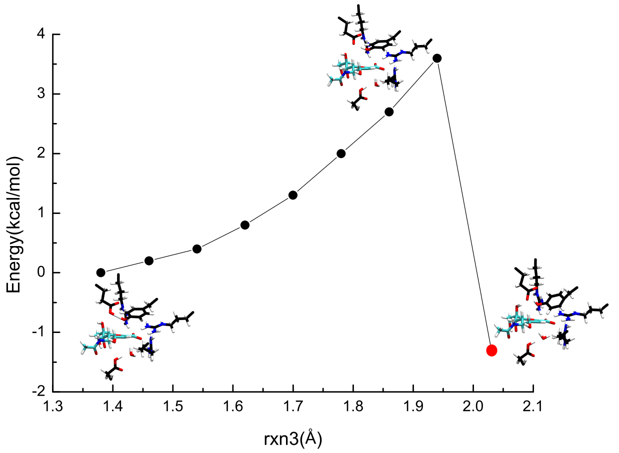

Figure 7: 1D potential energy plot (in kcal/mol) of the subsequent reaction for PathC in NanA. The last point (labelled in red) represents a free minimisation without restraints on the reaction coordinates to locate the PC state.

with the reaction coordinate rxn3=d5-d6. The results are shown in Fig. 6 and Fig. 7. For these two simple linear 1D maps, one can see that minor barriers were observed ($\approx$ 3.3 kcal/mol and $\approx$ 4.2 kcal/mol for PathB and PathC, respectively) to complete the whole process of reaction. As we described in the main context, NanA is capable of producing Neu5Ac along PathA with a relatively lower energies. These findings provide further evidence that PathA is the most likely reaction pathway for NanA.

7 MEP calculations for the second steps of NanB and NanC along the alternative reaction path of PathC with a catalytic water

The 2D MEP sampling on the second reaction steps along PathC for NanB and NanC based on the mechanisms (Fig. 8) that have a catalytic water molecule involved were evaluated with two predefined reaction coordinates, rxn3=d5-d6 and rxn7=d10-d12. d5 refers to the distance between the tyrosine Oη (Tyr653 and Tyr695 of NanB and NanC separately) and C2 position of the sugar ring, d6 corresponds to the one between the tyrosine Oη and Hη, d10 refers to the one between C3 and H32 in substrate, and d12 accounts for that between H32 of the sugar ring and the aspartic acid Oϵ2 (Asp270 and Asp315 for NanB and NanC, respectively) (Fig. 8). For NanB, rxn3 was harmonically restrained from -0.25 to 2.50 Å while rxn7 was scanned from -1.66 to 2.50 Å.For NanC, rxn3 was sampled from -0.19 to 2.45 Å while rxn7 was scanned from -3.17 to 2.59 Å. The scans were constructed in steps of 0.08 Åusing a force constant of 2,000.0 kcal/mol/Å^2. The contour plots of the energy profiles are illustrated in Fig. 9 and Fig. 10. The energy barrier for the two systems ($\approx$34.8 and 38.2 kcal/mol, respectively) is much higher than the one based on the mechanisms that without the catalytic water involved (Fig. 1 in the maintext and Fig. 3). These data further support the proposed mechanisms in this work but contradict with the previously proposed mechanisms.[6]

8 The extension MEP calculations for NanB and NanC to reach the final PC state

As the proton transfers between the tyrosine and the glutamic acid residue (NanB, Tyr653 and Glu541; NanC, Tyr695 and Glu584) involved in PathA, PathB and PathChave not been

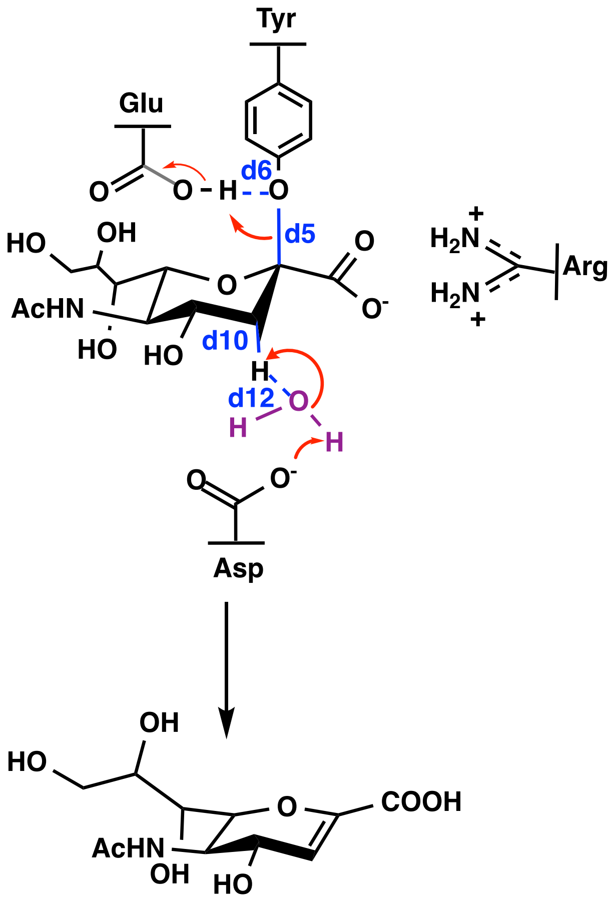

Figure 8: The alternative mechanism for the second step of NanB along PathC. Note there is a catalytic water involved.

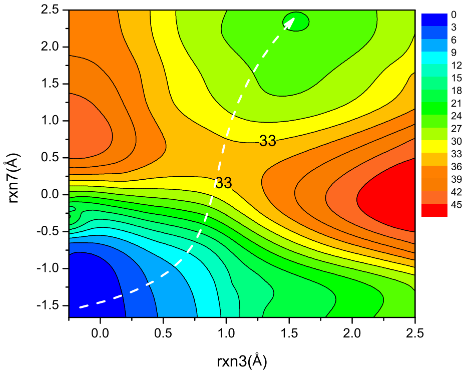

Figure 9: Potential energy contour plot for the second step of NanB based on the alternative mechanism of PathC which has a catalytic water involved. Energies are in kcal/mol.

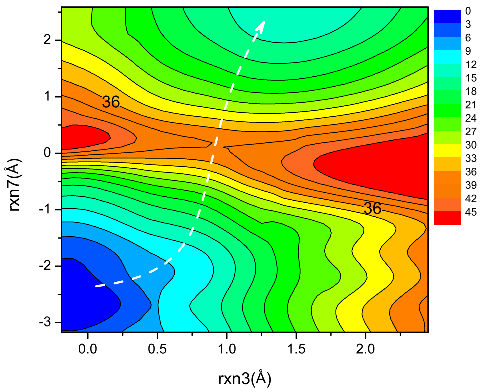

Figure 10: Potential energy contour plot for the second step of NanC based on the alternative mechanism which has a catalytic water involved. Energies are in kcal/mol.

completed during their PC states, we expanded the MEP calculations with the reaction coordinate rxn=d6. From the linear 1D maps (Fig. 11 to Fig. 16), one can see that minor barriers were observed (≈ 3.8, 2.3 and 1.8 kcal/mol for PathA, PathB and PathC of NanB, respectively; ≈ 2.3, 1.9 and 2.0 kcal/mol for PathA, PathB and PathC of NanC, respectively;) to complete the whole process of reaction (PC2).

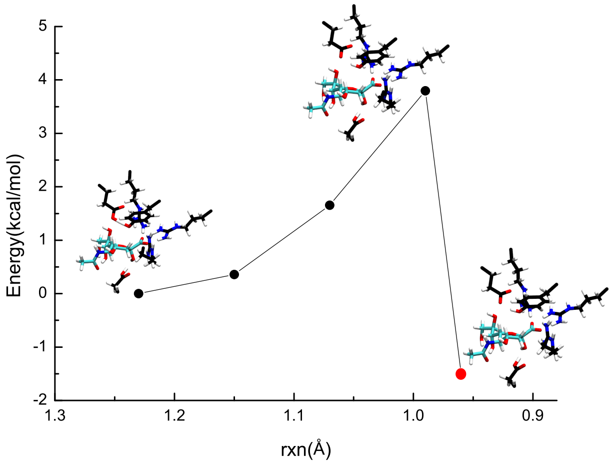

Figure 11: 1D potential energy plot (in kcal/mol) of the subsequent reaction for PathA in NanB. The last point (labelled in red) represents a free minimisation without restraints on the reaction coordinates to reach the final PC state.

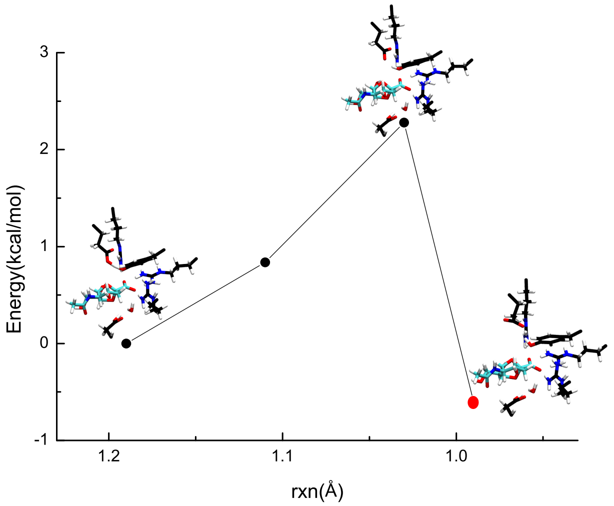

Figure 12: 1D potential energy plot (in kcal/mol) of the subsequent reaction for PathB in NanB. The last point (labelled in red) represents a free minimisation without restraints on the reaction coordinates to reach the final PC state.

9 Comparison of the mechanisms for NanA, NanB and NanC from IC to PC

The More O'Ferral-Jencks plots[8] for the PC formations of NanA along PathB and PathC, NanB along PathA, PathB and PathC, and NanC along PathA, PathB and PathC are shown from Fig. 17 to Fig. 19, where axes represent the Pauling bond orders from C2 at the axial position to thecatalytic water or the tyrosine residues (Tyr752 in NanA, Tyr653 in NanB and Tyr695 in NanC). Similar to the mechanisms of PathA of NanA, PathA of both NanB andNanC (Fig. 18 and Fig. 19) involve a dissociate $A_N D_N$ mechanism, in which the tyrosine Oη-sialyl bond is already cleaved while the O (water)-sialyl bond formation is beginning. Fig. 18 and Fig. 19 show that the tyrosine Oη-sialyl bond has been cleaved before the formation of the O7 (sialyl)-C2 (sialyl) bond is reached in PathB of NanA and NanC. Also, as depicted in Fig. 17, a proton has been transferred from the catalytic water to the Asp372 while the water molecule is protonated by HO7 of the sialyl cation duringthe reaction of PathB of NanA. However, there are no catalytic water molecules involved in PathB of NanB and NanC. Fig. 17 and Fig. 18 reveal that the tyrosine Oη-sialyl bond is cleaved before the

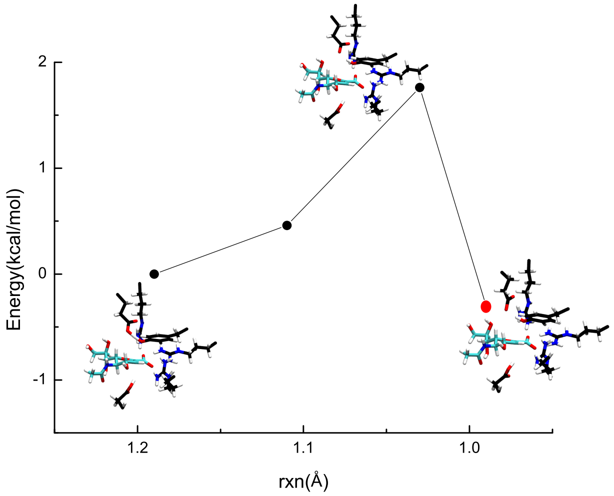

Figure 13: 1D potential energy plot (in kcal/mol) of the subsequent reaction for PathC in NanB. The last point (labelled in red) represents a free minimisation without restraints on the reaction coordinates to reach the final PC state.

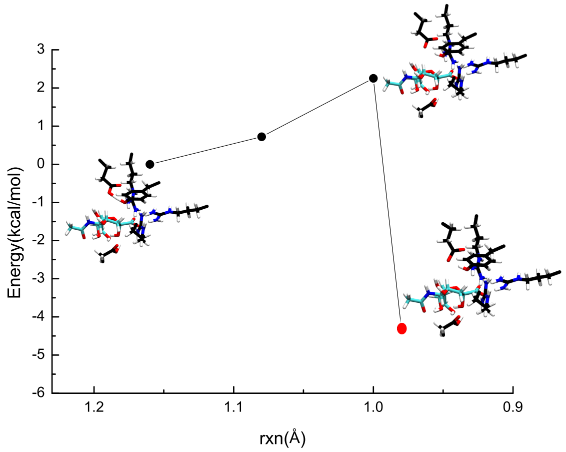

Figure 14: 1D potential energy plot (in kcal/mol) of the subsequent reaction for PathA in NanC. The last point (labelled in red) represents a free minimisation without restraints on the reaction coordinates to reach the final state.

the TS2 is reached in PathC of both NanA and NanB. At the same time, the proton transfer between the catalytic water molecule and the Asp372 of NanA is about to happen, and the proton transfer between Asp270 in NanB and H32 of the sialyl cation hashappened.

The values for the distance at the critical points of the preferred pathways of the three sialidases (PathA of NanA, PathB of NanB and PathC of NanC) are presented in Table 3 to 8. At the TS1, the proton transfer between the tyrosine and the glutamic acid residue is

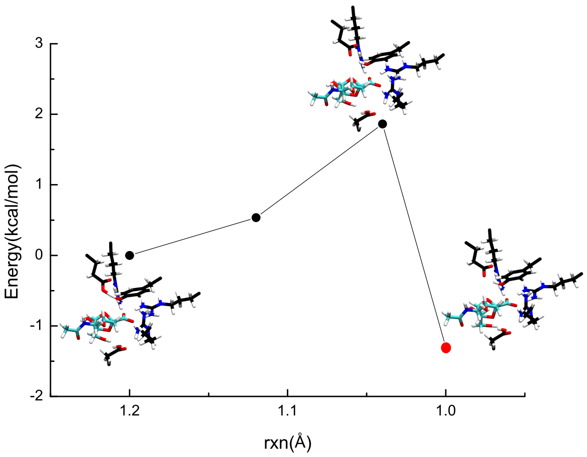

Figure 15: 1D potential energy plot (in kcal/mol) of the subsequent reaction for PathB in NanC. The last point (labelled in red) represents a free minimisation without restraints on the reaction coordinates to reach the final PC sate.

almost completed (d4≈1.18, 1.14 and 1.21 Å, respectively). All Tyr752, Tyr653 and Tyr695 in the three cases are more negatively charged, that enhances them being a nucleophile (See Chapter3 for more information). The hydroxyl oxygen of the nucleophilic tyrosine is moving closer to the C2 position of the substrate, the distance between which is 2.24 Å in NanB which is the shortest one among the three cases (d3≈2.40 and 2.64 Å for NanA and NanC, respectively), making Tyr653 of NanB a better nucleophile. The tyrosine Oη-sialyl is already cleaved (d1≈2.07, 2.24 and 2.10 Å, respectively). Additionally, the proton transfer between the aspartic acid residue and the methyl group has been completed in NanB and NanC (d2≈1.05 Å and 1.02 Å, respectively), except in NanA (d2≈1.22 Å, See the main article for more information). Therefore, the reaction energy barrier of the first step of NanB is the lowest one (See the maintext for more information).

At the TS2, the tyrosine Hη-Oη bond formation is beginning in NanA and NanC (d6≈1.15 and 1.23 Å, respectively), while the Tyr653 Hη still binds with Glu541 Oϵ instead of moving towards to the Tyr653 Oη in NanB (d6≈1.59 Å). In the meantime, the sialyl cation O7 is ∼1.95 Å closer to the C2 position of the substrate in NanB while it moves slower in NanA (∼1.15 Å closer to the anomeric C2). The cleavage of sialyl cation C3-H32 bond and the formation of sialyl cation H32-Asp315 have already been completed in NanC (d10≈1.59 Å

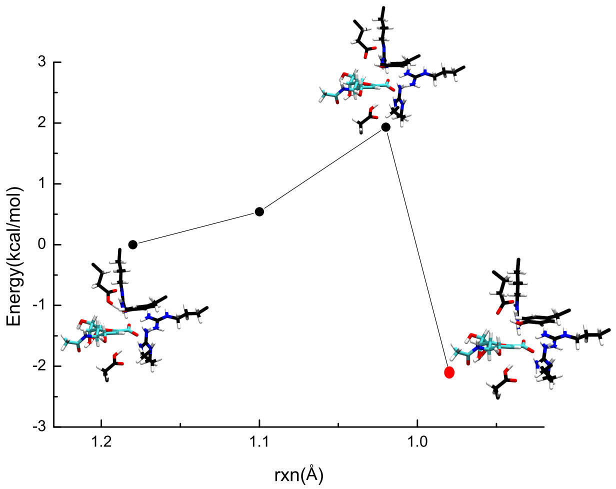

Figure 16: 1D potential energy plot (in kcal/mol) of the subsequent reaction for PathC in NanC. The last point (labelled in red) represents a free minimisation without restraints on the reaction coordinates to reach the final PC state.

and d11≈1.05 Å, See the main article for more information). With these in mind, NanC reached its TS2 first, followed by NanB and NanA, respectively. This is consistent with the MEP results.

Table 3: Reaction coordinate distances (Å) for RC, TS1 and IC in the first step of NanA.

	RC	TS1	IC
d1	1.50	2.07	3.47
d2	1.62	1.22	1.03
d3	3.91	2.40	1.54
d4	1.68	1.18	1.00

Table 4: Reaction coordinate distances (Å) for RC, TS1 and IC in the first step of NanB.

	RC	TS1	IC
d1	1.52	2.24	3.53
d2	1.65	1.05	1.02
d3	2.99	2.24	1.57
d4	1.27	1.14	1.01

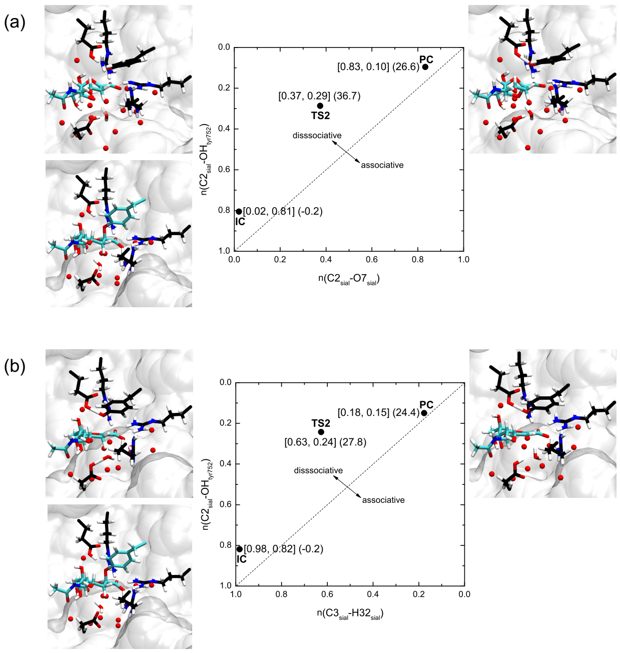

Figure 17: (a) The More O'Ferral-Jencks plot for the PC formation in PathB of NanA. The axes are the Pauling bond orders from the anomeric C to the O-7 atom of the substrate (n($C2_{sial}$-$O7_{sial}$)) and residue Tyr752 (n($C2_{sial}$-OH_{tyr752})); (b) The More O'Ferral-Jencks plot for the PC formation in PathC of NanA. The axes are the Pauling bond orders from the anomeric C to the H-3 atom of the substrate (n($C2_{sial}$-$H32_{sial}$)) and residue Tyr752 (n($C2_{sial}$-OH_{tyr752})). The IC, TS2, and PC configuration are shown in the bottom left, top left, and top right, respectively. The catalytic water molecule is shown in Licorice and the nearby water molecules are presented in red with CPK.

10 Free energy profiles for NanA along PathB and PathC

Fig. 20 show the free energy contour plots for the PathB and PathC from the IC to the PC. The minimum free energy paths highlighted with white dashed lines show similar trend with MEP, indicating that the MEP calculations can describe the catalytic mechanisms of NanA properly. These free energy profiles canbe compared with that for NanA along PathA in Fig. 7 in the maintext.

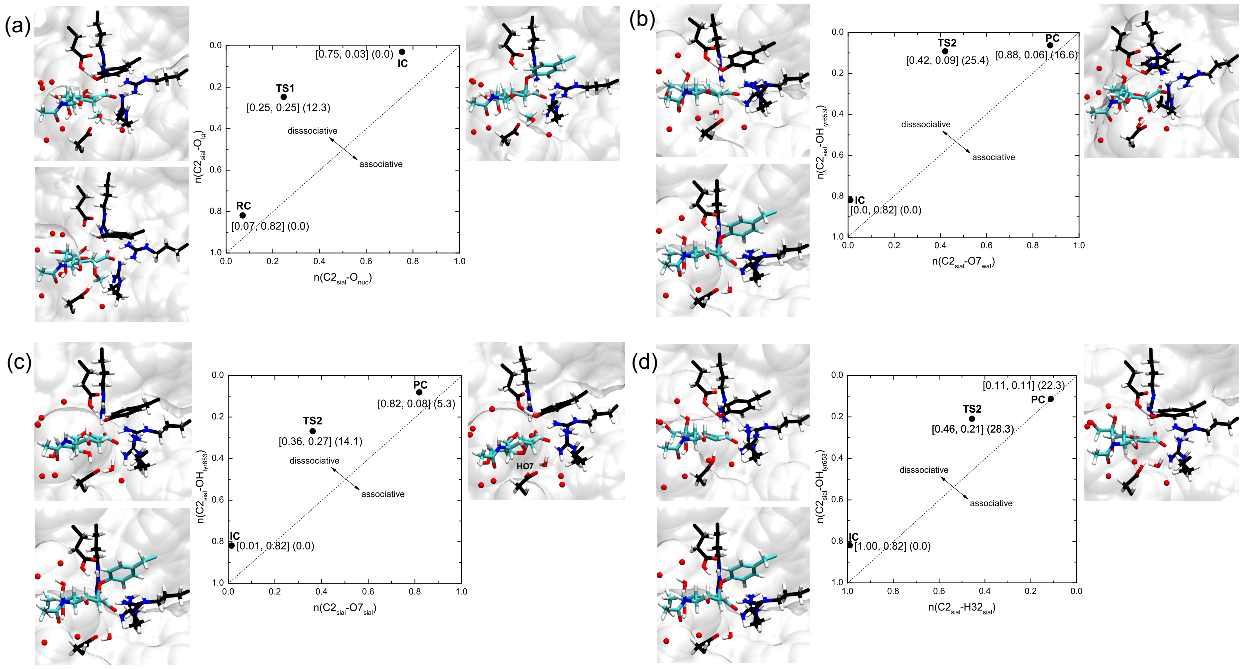

Figure 18: (a) The More O'Ferral-Jencks plot for the IC formation of NanB. The axes are the Pauling bond orders from the anomeric C to the nucleophile (n($C2_{sial}$-O_{nuc})) and the leaving group (n($C2_{sial}$-O_{lg})); (b) The More O'Ferral-Jencks plot for the PC formation in PathA of NanB. The axes are the Pauling bond orders from the anomeric C to the water molecule (n($C2_{sial}$-O_{wat})) and residue Tyr653(n($C2_{sial}$-OH_{tyr653})); (c) The More O'Ferral-Jencks plot for the PC formation in PathB of NanB. The axes are the Pauling bond orders from the anomeric C to the O-7 atom of the substrate (n($C2_{sial}$-$O7_{sial}$)) and residue Tyr653 (n($C2_{sial}$-OH_{tyr653})); (d) The More O'Ferral-Jencks plot for the PC formation in PathC of NanB. The axes are the Pauling bond orders from the anomeric C to the H-3 atom of the substrate (n($C2_{sial}$-$H32_{sial}$)) and residue Tyr653 (n($C2_{sial}$-OH_{tyr653})). The RC, TS1, IC, and IC, TS2, and PC configuration are shown in the bottom left, top left, and top right, respectively. The catalytic water molecule is shown in Licorice and the nearby water molecules are presented in red with CPK.

11 Perturbative analyses on the second step of PathA in NanA

Here, to probe the effects of different active site residues among NanA, NanB and NanC on the reaction barrier for the second step of PathA in NanA, we carried out perturbative analyses on the IC and TS2. Particularly we were interested in the residues which are different among NanA, NanB and NanC. In the perturbative analysis, the partial charges on the specific residue of interest were set to zero and then the energies were recalculated based

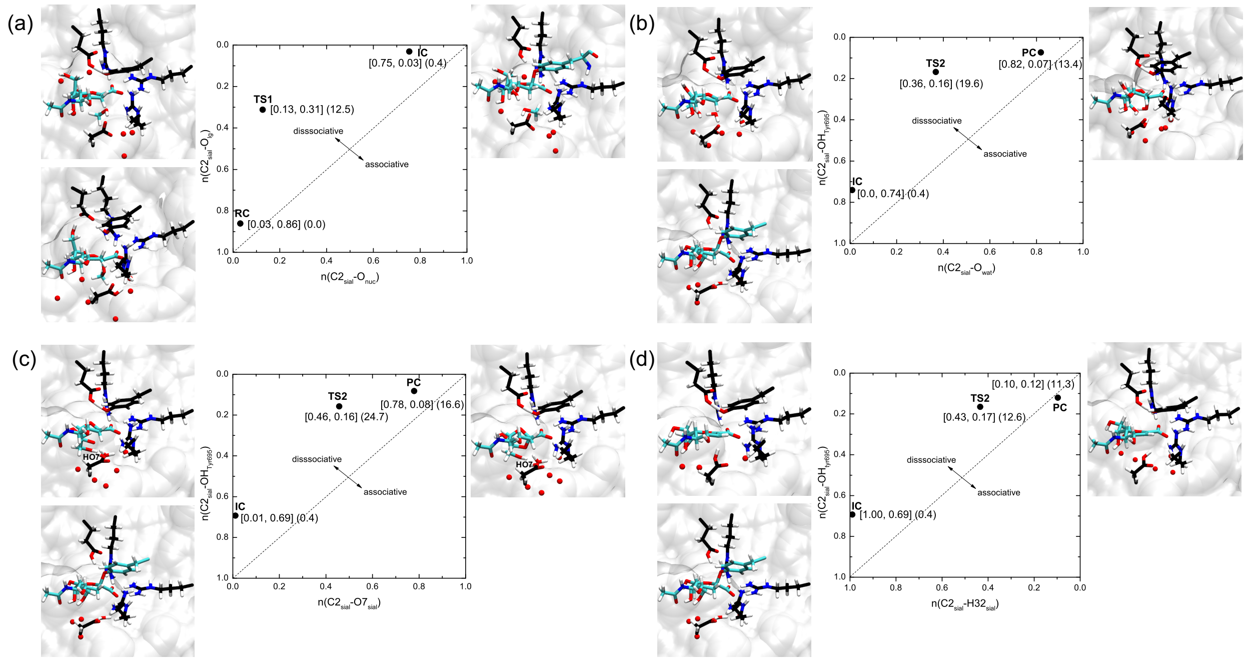

Figure 19: (a) The More O'Ferral-Jencks plot for the IC formation of NanC. The axes are the Pauling bond orders from the anomeric C to the nucleophile ($n(C2_{sial}$-$O_{nuc})$) and the leaving group ($n(C2_{sial}$-$O_{lg})$); (b) The More O'Ferral-Jencks plot for the PC formation in PathA of NanC. The axes are the Pauling bond orders from the anomeric C to the water molecule ($n(C2_{sial}$-$O_{wat})$) and residue Tyr695 ($n(C2_{sial}$-$OH_{tyr695})$); (c) The More O'Ferral-Jencks plot for the PC formation in PathB of NanC. The axes are the Pauling bond orders from the anomeric C to the O-7 atom of the substrate ($n(C2_{sial}$-$O7_{sial})$) and residue Tyr695 ($n(C2_{sial}$-$OH_{tyr695})$); (d) The More O'Ferral-Jencks plot for the PC formation in PathC of NanC. The axes are the Pauling bond orders from the anomeric C tothe H-3 atom of the substrate ($n(C2_{sial}$-$H32_{sial})$) and residue Tyr695 ($n(C2_{sial}$-$OH_{tyr695})$). The RC, TS1, IC, and IC, TS2, and PC configuration are shown in the bottom left, top left, and top right, respectively. The catalytic water molecule is shown in Licorice and the nearby water molecules are presented in red with CPK.

on the optimised geometries of the reference state (the wild type sequence of NanA) from the MEP simulations. The obtained reaction barriers can be compared to that for the reference state. Sucha perturbative analysis can provide qualitative information on the contributions of relative residues, albeit the relaxation of the modified environment is neglected.[7,9] The results from the perturbative analyses are presented in Table. 9. To our surprise, in all the cases, the resultant values for the energy barriers are relatively similar to the reference value for PathA in NanA, indicating that the stabilisation effect of these residues are minor. It is worth noting that these residues are relatively far away from the reactive centre.

Table 5: Reaction coordinate distances (Å) for RC, TS1 and IC in the first step of NanC.

	RC	TS1	IC
d1	1.49	2.10	3.49
d2	2.34	1.02	0.98
d3	3.49	2.64	1.57
d4	1.24	1.21	1.00

Table 6: Reaction coordinate distances (Å) for IC, TS2 and PC for the second step of NanA along PathA, PathB and PathC, respectively.

		IC	TS2	PC
PathA	d5	1.52	2.63	3.05
	d6	1.95	1.15	1.09
	d7	3.26	2.11	1.50
	d8	1.79	1.66	1.01
PathB	d5	1.53	2.37	2.80
	d6	1.81	1.69	1.16
	d9	3.83	1.99	1.51
PathC	d5	1.52	2.24	2.54
	d6	1.82	1.20	1.16
	d10	1.12	1.37	2.13
	d11	1.89	1.17	0.98

12 Water distribution in the active sites of NanA, NanB and NanC

As proposed in the previous study,[10] there exists a noticeable difference in local environment around the catalytic site. We further hypothesised that the solvation environment might play a more significant role on the reaction energetics. So we charaterised the water contributions from the SCC-DFTB/MM simulations of IC for NanA, NanB and NanC by computing the water radial distribution functions around the C2, HO7 and H32 position of sialic acid of NanA, NanB and NanC, respectively (Fig. 21). For NanA, the overall values for the coordination number for the water molecules around C2 position of the substrateare higher than those for NanB and NanC, which means the reaction catalyzed by NanA make the C2 of the sugar more exposed to water, and this is consistent with the hypothesis that

Table 7: Reaction coordinate distances (Å) for IC, TS2 and PC for the second step of NanB along PathA, PathB and PathC, respectively.

		IC	TS2	PC
PathA	d5	1.52	2.83	3.06
	d6	1.66	1.21	1.20
	d7	4.58	1.92	1.48
	d8	2.08	1.39	0.98
PathB	d5	1.52	2.19	2.91
	d6	1.64	1.59	1.19
	d9	3.96	2.01	1.52
PathC	d5	1.52	2.34	2.71
	d6	1.77	1.31	1.19
	d10	1.09	1.56	2.40
	d11	3.04	1.10	0.99

Table 8: Reaction coordinate distances (Å) for IC, TS2 and PC for the second step of NanC along PathA, PathB and PathC, respectively.

		IC	TS2	PC
PathA	d5	1.58	2.47	2.97
	d6	1.61	1.22	1.16
	d7	5.06	2.00	1.52
	d8	1.81	1.51	1.10
PathB	d5	1.62	2.51	2.90
	d6	1.81	1.34	1.20
	d9	4.12	1.87	1.55
PathC	d5	1.62	2.48	2.67
	d6	1.81	1.23	1.18
	d10	1.09	1.59	2.49
	d11	2.92	1.05	0.98

the water molecule can easily attack C2 position along PathA in NanA.[10] In other words, the influence of activated water molecule was supposed to have significant impacts on the reaction mechanism of NanA, and PathA is the preferred reaction pathway for NanA.

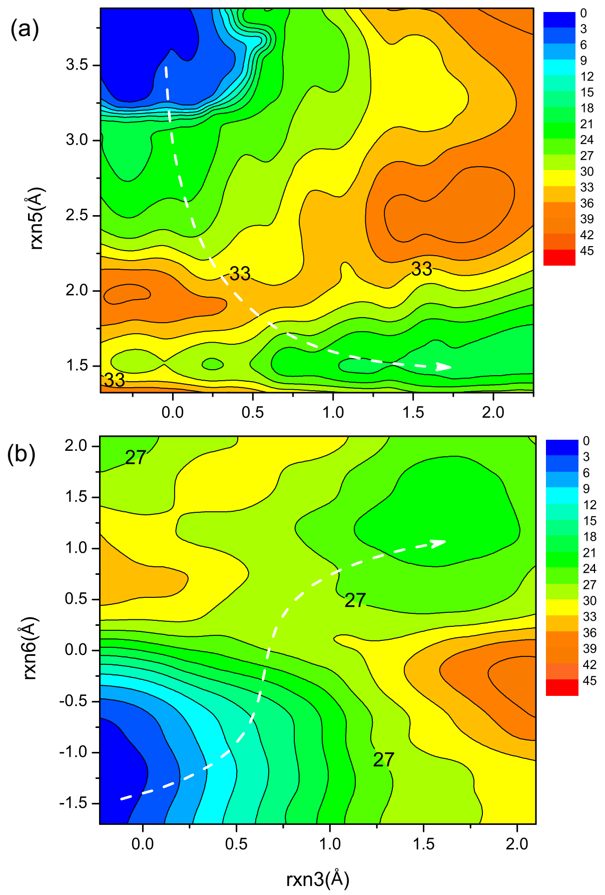

Figure 20: Free energy contour plot (in kcal/mol) for (a) PathB and (b) PathC of NanA. Energies are in kcal/mol. The white dashed line illustrates the minimum free energy path.

Table 9: Potential energies based on perturbation analyses of relevant residues in NanA with SCC-DFTB/MM simulations. Energies are in kcal/mol.

		IC	TS2
Different residues	WT	0.0	27.0
	Gln602	0.0	27.7
	Tyr695	0.0	27.7
	Asn645	0.0	27.8
	Tyr777	0.0	28.2
	Phe443	0.0	26.7

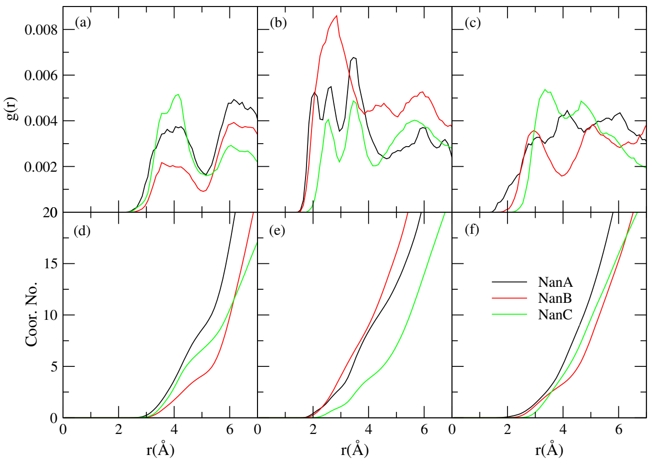

Figure 21: Water distribution around C2, HO7 and H32 in substrate from the SCC-DFTB/MM simulations for NanA, NanB and NanC (a) Radial distribution functionof water oxygens around C2 in substrate in intermediate state of NanA, NanB and NanC; (b) Radial distribution function of water oxygens around HO7 in substrate in intermediate state of NanA, NanB and NanC; (c) Radial distribution function of water oxygens around H32 in substrate in intermediate state of NanA, NanB and NanC; (d) the coordination number for water around around C2 in substrate in intermediate state of NanA, NanB and NanC; (e) the coordination number for water around around HO7 in substrate in intermediate state of NanA, NanB and NanC; (f) the coordination number for water around around H32 in substrate in intermediate state of NanA, NanB and NanC.

References

(1) Schaefer, P.; Riccardi, D.; Cui, Q. Reliable treatment of electrostatics in combined QM/MM simulation of macromolecules. *The Journal of Chemical Physics* **2005**, *123*, 014905.

(2) Cremer, D.; Pople, J. General definition of ring puckering coordinates. *Journal of the American Chemical Society* **1975**, *97*, 1354–1358.

(3) Montgomery, A. P.; Xiao, K.; Wang, X.; Skropeta, D.; Yu, H. Computational Glycobiology: Mechanistic Studies of Carbohydrate-Active Enzymes and Implication for Inhibitor Design. *Advances in Protein Chemistry and Structural Biology* **2017**, *109*, 25–76.

(4) Xu, G.; Li, X.; Andrew, P. W.; Taylor, G. L. Structure of the catalytic domain of *Streptococcus pneumoniae* sialidase NanA. *Acta Crystallographica Section F: Structural Biology and Crystallization Communications* **2008**, *64*, 772–775.

(5) Xu, G.; Potter, J. A.; Russell, R. J.; Oggioni, M. R.; Andrew, P. W.; Taylor, G. L. Crystal structure of the NanB sialidase from *Streptococcus pneumoniae. Journal of Molecular Biology* **2008**, *384*, 436–449.

(6) Owen, C. D.; Lukacik, P.; Potter, J. A.; Sleator, O.; Taylor, G. L.; Walsh, M. A. *Streptococcus pneumoniae* NanC structural insights into the specificity and mechanism of a sialidase that produce a silidase inhibitor. *Journal of Biological Chemistry* **2015**, *290*, 27736–27748.

(7) Yu, H.; Griffiths, T. M. pK_a cycling of the general acid/base in glycoside hydrolase families 33 and 34. *Physical Chemistry Chemical Physics* **2014**, *16*, 5785–5792.

(8) Guthrie, R. D.; Jencks, W. P. IUPAC recommendations for the representation of reaction mechanisms. *Accounts of Chemical Research* **1989**, *22*, 343–349.

(9) Xiao, K.; Yu, H. Rationalising pK_a shifts in *Bacillus circulans* xylanase with computational studies. *Physical Chemistry Chemical Physics* **2016**, *18*, 30305–30312.

(10) Xu, G.; Kiefel, M. J.; Wilson, J. C.; Andrew, P. W.; Oggioni, M. R.; Taylor, G. L. Three *Streptococcus pneumoniae* sialidases: Three different products. *Journal of the American Chemical Society* **2011**, *133*, 1718–1721.
